# Trifluoromethyl‐Substituted Conjugated Random Terpolymers Enable High‐Performance Small and Large‐Area Organic Solar Cells Using Halogen‐Free Solvent

**DOI:** 10.1002/advs.202302376

**Published:** 2023-06-25

**Authors:** Zia Ur Rehman, Muhammad Haris, Seung Un Ryu, Muhammad Jahankhan, Chang Eun Song, Hang Ken Lee, Sang Kyu Lee, Won Suk Shin, Taiho Park, Jong‐Cheol Lee

**Affiliations:** ^1^ Advanced Energy Materials Research Center Korea Research Institute of Chemical Technology (KRICT) Daejeon 34114 Republic of Korea; ^2^ Advanced Materials and Chemical Engineering University of Science and Technology (UST) Daejeon 34113 Republic of Korea; ^3^ Department of Chemical Engineering Pohang University of Science and Technology (POSTECH) Gyeongsangbuk‐do Pohang 37673 Republic of Korea

**Keywords:** halogen‐free solvent, organic solar cells, random copolymerization, sub‐module devices, trifluoromethyl‐substitution

## Abstract

The advancement of non‐fullerene acceptors with crescent‐shaped geometry has led to the need for polymer donor improvements. Additionally, there is potential to enhance the photovoltaic parameters in high‐efficiency organic solar cells (OSCs). The random copolymerization method is a straightforward and effective strategy to further optimize photoactive morphology and enhance device performance. However, finding a suitable third component in terpolymers remains a crucial challenge. In this study, a series of terpolymer donors (PTF3, PTF5, PTF10, PTF20, and PTF50) is synthesized by introducing varying amounts of the trifluoromethyl‐substituted unit (CF3) into the PM6 polymer backbone. Even subtle changes in the CF3 content can significantly enhance all the photovoltaic parameters due to the optimized energy levels, molecular aggregation/miscibility, and bulk‐heterojunction morphology of the photoactive materials. Thus, the best binary OSC based on the PTF5:Y6‐BO achieves an outstanding power conversion efficiency (PCE) of 18.2% in the unit cell and a PCE of 11.6% in the sub‐module device (aperture size: 54.45 cm^2^), when using halogen‐free solvent *o*‐xylene. This work showcases the remarkable potential of the easily accessible CF3 unit as a key constituent in the construction of terpolymer donors in high‐performance OSCs.

## Graphical abstract

A series of random terpolymers is developed by incorporating readily accessible trifluoromethyl‐substituted benzene (TFB) units into the PM6 donor polymer. Incorporation of the TFB unit leads to optimal molecular packing and desirable aggregation/miscibility morphology in halogen‐free solvents, resulting in substantial efficiencies of 18.2% and 11.6% in the unit cell and sub‐module devices.

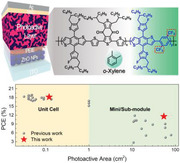

## 1 Introduction

Organic solar cells (OSCs) have received a great deal of attention from both researchers and the industry due to their unique properties, such as mechanical flexibility, light weight, and solution‐processable fabrication. In addition, OSCs have potential in the future energy marketplace because of their scalability and low energy payback time.^[^ ^1^, ^2^, ^3^, ^4^, ^5^ ^]^ Over the past few decades, advancements in organic photoactive materials and device engineering processes have led to a continuous enhancement in power conversion efficiency (PCE). As a result, the PCE has increased significantly from 7% to 19%.^[^ ^6^, ^7^, ^8^, ^9^, ^10^, ^11^, ^12^, ^13^ ^]^ Notable progress has been made in the development of high‐performance non‐fullerene acceptors (NFAs), such as ITIC, Y6, and their derivatives.^[^ ^14^, ^15^, ^16^, ^17^ ^]^ However, several crucial requirements must be met to facilitate the transition of OSCs from laboratory research to viable commercialization. To successfully realize the practical use of OSCs, it is essential for high‐efficiency devices with excellent thickness tolerance, long device lifetime under working conditions, eco‐friendly solvent processes, minimized cell‐to‐module loss, and simple scalability of material production with batch‐to‐batch reproducibility and molecular weight insensitivity.^[^ ^18^ ^]^ Therefore, developing novel photoactive materials that can meet the aforementioned parameters is crucial.

Currently, the wide‐bandgap D‐A copolymer PM6 (BDT‐F as D‐unit and BDD as A‐unit) is considered as the most representative high‐performance polymer donor for Y6‐based OSCs.^[^ ^19^ ^]^ Although OSCs based on PM6:Y6 exhibit a high PCE of over 15%, the synthetic cost of PM6 is high, mostly due to the complex and multi‐stage synthesis of both the D/A‐unit; therefore, lowering the synthetic cost of PM6 and further enhancing its photovoltaic efficiency are of considerable significance. To improve the photovoltaic performance of cost‐effective PM6 polymer derivatives without sacrificing optoelectronic properties, random copolymerization has emerged as a promising synthetic strategy. In this approach, a third component is introduced into the D‐A backbone of PM6 polymers, such as the D1‐A‐D2‐A type that incorporates another electron‐donating unit (D2) or the D‐A1‐D‐A2 type that includes another electron‐accepting unit (A2).^[^ ^12^ ^]^ The electronic energy levels, optical properties, and molecular configuration of the terpolymers can be effectively fine‐tuned by introducing the third component while still maintaining the desired bulk‐heterojunction (BHJ) morphology and having adequate batch‐to‐batch reproducibility of the photoactive materials. For example, Hou et al. introduced an ester‐substituted thiophene (EST) as the A2 unit into the PM6 backbone, resulting in a terpolymer with broadened optical absorption and downshifted electronic energy levels.^[^ ^20^ ^]^ A PCE of over 15% was achieved by using a T1 terpolymer with 20% proportion of EST as the donor, and IT‐4F as the acceptor. Zhang et al. incorporated thiophene‐thiazolothiazole (TTZ) as an A2‐unit with 20% proportion into the PM6 polymer backbone and obtained D‐A1‐D‐A2‐type random terpolymers (known as PM1), exhibiting a lower highest occupied molecular orbital (HOMO) energy level, higher hole mobility, and more favorable morphology. The PM1:Y6‐based OSC achieved a high PCE of 17.6%.^[^ ^21^ ^]^ Similarly, Guo et al. incorporated 20% of the 5,5′‐dithienyl‐2,2′‐bithiazole (DTBTz) as an A2‐unit into the backbone of PM6, resulting in a new terpolymer referred to as PM6‐Tz20, which can effectively tailor molecular orientation and aggregation as well as optimize photoactive morphology.^[^ ^22^ ^]^ As a result, the OSC based on PM6‐Tz20:Y6 attained a 17.6% PCE. However, the most successful demonstration of high‐efficiency OSCs based on terpolymer donors has relied on halogenated solvent systems such as chloroform and chlorobenzene, with halogenated solvent additive (1‐chloronaphthalene or 1,8‐diiodoctane)^[^ ^12^, ^20^, ^21^, ^22^ ^]^ to achieve sufficient solubility of the photoactive materials and the creation of an ideal morphology. The use of these halogenated solvent systems is problematic because of their toxicity and potential harm to both humans and the environment. Moreover, the incorporated third components are typically terpolymers that are difficult and expensive to synthesize.^[^ ^23^, ^24^ ^]^ Therefore, modifying the terpolymer donors to produce large‐area OSCs that are both environmentally friendly and highly efficient in non‐halogenated solvents is required.

In this study, we selected a strong electron withdrawing group (1,4‐bis(trifluoromethyl)benzene, TFB), an easy commercially assessable monomer, to develop high‐performance OSCs based on PM6‐based terpolymers, which can be processed from non‐halogenated solvent and applied to large‐area applications. Although trifluoromethylation is often used to change the biological qualities of bioavailability, basicity, lipophilicity, and metabolic stability in bioactive compounds,^[^ ^25^ ^]^ this approach has been rarely studied in OSCs.^[^ ^26^, ^27^ ^]^ One of the strongest electron‐withdrawing groups in structural organic chemistry is the CF3 group with an electronegativity value of 3.5 (Pauling scale). When connected to C (sp^2^) atoms, they form stronger electron‐withdrawing groups than F atoms. As a result, CF3 units can establish non‐covalent interactions (C—F∙∙∙H, F∙∙∙S, and C—F∙∙∙*π* interactions) even better than an F atom. Therefore, the trifluoromethyl benzene (TFB) can promote greater intramolecular charge transfer (ICT) from the BDT groups than the BDD unit in PM6 owing to the significant electrostatic effect and electron deficiency of trifluoromethyl groups, resulting in slightly red‐shifted absorption. Furthermore, trifluoromethylated compounds exhibit greater polarization and reduced coulombic potential between electrons and holes, resulting in improved dissociation of excitons and subsequently leading to an enhanced short‐circuit current density (*J*_SC_) value in OSCs.^[^ ^28^ ^]^ Moreover, the strong electron‐withdrawing nature of the CF3 group can downshift HOMO energy levels, which is advantageous for the enhancement of open‐circuit voltage (*V*_OC_). The high electron dipole of CF3 substitution is well recognized for inducing intermolecular aggregation, which enhances the molecular packing and BHJ morphology. In addition, the lipophilic nature of the CF3 group will increase the solubility of the terpolymer in non‐polar solvents such as toluene and *o*‐xylene.

In this study, we developed a series of PM6‐based terpolymers by gradually replacing the BDD groups with the TFB to augment the optical absorption, electronic energy level, molecular stacking, and ultimately the photovoltaic performances. These novel terpolymers, namely PTF3, PTF5, PTF10, PTF20, and PTF50, contained 3%, 5%, 10%, 20%, and 50% TFB content, respectively. Through a morphological analysis on the photoactive materials, we found that the PTF5:Y6‐BO exhibits desirable and well‐controlled BHJ morphology, ascribed to the appropriate regulation of the miscibility and aggregation between the polymer donor and non‐fullerene acceptor. This favorable interpenetrating network with optimized phase separation is beneficial to the efficient charge transfer/extraction along with suppressed carrier recombination. Finally, the champion PTF5:Y6‐BO‐based OSC achieved an excellent PCE of 18.2% with a *V*_OC_ of 0.84 V, *J*_SC_ of 28.0 mA cm^−2^, and fill factor (FF) of 77.2% on a scale of 0.12 cm^2^ photoactive area, which is much higher than that of the control device based on the PM6:Y6‐BO system (PCE = 15.1%, *V*_OC_ = 0.82 V, *J*_SC_ = 25.8 mA cm^−2^, and FF = 71.3%), when processed in halogen‐free solvents. Furthermore, this promising PTF5:Y6‐BO photoactive material is applied to large‐area sub‐module devices to corroborate the importance of scalability in OSCs. The best PCE of 11.6% (certified 11.5%) with a 54.45 cm^2^ photoactive area was obtained, setting a record of binary OSCs processed based on halogen‐free solvents. These results indicate that a random copolymerization strategy based on the CF3 unit holds immense promise as an effective approach for enhancing the photovoltaic performance of OSCs.

## 2 Results and Discussion

**Figure 1:**
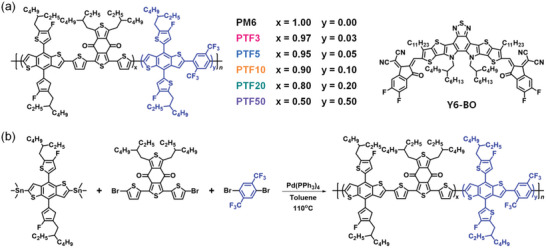
a) Molecular structures of polymer donors (PM6 and terpolymers) and Y6‐BO. b) Polymerization route of the terpolymers.

Terpolymers were synthesized by standard Stille coupling reactions in toluene with Pd(PPh_3_)_4_ acting as a catalyst for 24 h at 110 °C by varying the amount of TFB units (3–50%) added to the reaction mixture. Then, by using the Soxhlet extraction method (extraction with methanol, acetone, *n*‐hexane, and chloroform), the random copolymers were purified and dried under a vacuum for 12 h after being precipitated into methanol. After being fully purified, the copolymer yields were over 60%. The chemical structure and synthetic route of the terpolymer donors are shown in **Figure** **1** and the detailed synthetic conditions are described in the Supporting Information. Every terpolymer donor exhibited strong solubility in typical organic solvents such as chloroform, chlorobenzene, toluene, and *o*‐xylene, and their thermal properties are presented in Figure S1, Supporting Information. According to a thermal gravimetric analysis (TGA), the decomposition temperature, which was determined by a weight loss of 5%, was calculated to be over 400 °C for PM6, PTF3, PTF5, PTF10, and PTF20; however, PTF50 showed a decomposition temperature below 400 °C. These polymers, therefore, have sufficient heat resistance and up to 20% insertion of TFB into the polymer backbone to avoid altering the molecular thermal stability. Moreover, differential scanning calorimetry (DSC) thermograms show that the PM6 and terpolymers have similar thermal properties without any noticeable exothermic or endothermic peaks. Under ambient conditions, the obvious molecular melting point below the decomposition temperature was not found.

**Figure 2:**
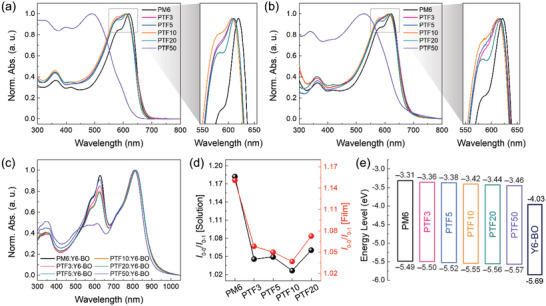
UV–vis absorption spectra of polymer donors in a) solution and b) thin film states processed from *o*‐xylene. c) UV–vis absorption spectra of polymer donor:Y6‐BO blend films. d) *I*_0‐0_/*I*_0‐1_ ratio in solution and thin film states for PM6 and terpolymers. e) Energy level diagrams of polymer donors and Y6‐BO acceptor.

UV–vis absorption measurements were executed to detect the impact of CF3 content on the optical characteristics of terpolymer donors, as illustrated in **Figure** **2**, and their optical properties are tabulated in **Table** **1**. Figure 2 provides the normalized absorption spectra of the polymers in a solution and thin films, along with the pertinent optical characteristics (Figure 2a,b). Strong absorptions between 400 and 650 nm were visible in the films of the synthetic polymers, and they formed an excellent complementary absorption with the Y6‐BO acceptor (Figure 2c). The *E*_g_ determined from the optical absorption onsets is also comparable to one another, except for PTF50, and are as follows: 1.84, 1.82, 1.83, 1.83, 1.85, and 1.89 eV for PM6, PTF3, PTF5, PTF10, PTF20, and PTF50, respectively. The maximum absorption peaks of the synthesized polymers were slightly blue shifted both in the solution and film states compared with PM6, certainly implying randomized terpolymer backbones.^[^ ^29^, ^30^ ^]^ However, the slightly red shifted absorption edge of the terpolymers is attributed to enhanced ICT effects between the BDT‐F unit and the introduced third CF3 component.^[^ ^31^ ^]^ As a result, the inclusion of the TFB unit induces a more suitable and linear packing structure. In addition, compared to PM6, the TFB‐containing terpolymer had a lower *I*_0‐0_/*I*_0‐1_ transition peak ratio (Figure 2d), indicating an obvious alleviation of aggregation characteristics under the *o*‐xylene solvent.^[^ ^32^, ^33^, ^34^ ^]^ Upon further increasing the CF3 unit, different absorbance characteristics appear with a 50% TFB component in both the solution and film absorption spectra. These changes are verified by the diminished intermolecular stacking and push‐pull effect and the aggregation destroyed by the random terpolymerization strategy upon excessive inclusion of the third component. Thus, it is crucial to regulate the CF3 content up to a certain level in the terpolymers for effective intermolecular interactions.

**Table 1:** Molecular weight, thermal, optical, and electrochemical properties of the polymer donors

Polymer	*M*_n_ [kDa]	PDI	*T*_d_ [°C]^a)^	*λ*_max_^sol^ [nm]	*λ*_max_^film^ [nm]	*E*_g_^opt^ [eV]^b)^	HOMO [eV]^c)^	LUMO [eV]^c)^	LUMO [eV]^d)^
PM6	159	3.51	438	619	622	1.84	‒5.49	‒3.31	‒3.65
PTF3	168	4.38	436	609	616	1.82	‒5.50	‒3.36	‒3.66
PTF5	158	4.04	439	609	615	1.83	‒5.52	‒3.38	‒3.71
PTF10	148	5.00	436	607	613	1.83	‒5.55	‒3.42	‒3.72
PTF20	140	4.20	461	611	617	1.85	‒5.56	‒3.44	‒3.71
PTF50	126	4.15	373	489	528	1.89	‒5.57	‒3.46	‒3.69

^a)^ Decomposition temperature (*T*_d_) was determined by TGA (with 5% weight loss);

^b)^ Estimated values from the UV–vis absorption edge of the thin films;

^c)^ Electrochemically determined versus Fc/Fc^+^, HOMO energy levels estimated from the onset oxidation, assuming the absolute Energy level of Fc/Fc^+^ to be 4.8 eV below vacuum;

^d)^ Calculated from HOMO energy levels and *E*_g_^opt^.

By using electrochemical cyclic voltammetry (CV), the energy levels of the polymers were calculated, and their electrochemical characteristics are shown in Figure S2, Supporting Information. From the onset of the oxidation potential curves, which were calibrated by the ferrocene/ferrocenium (Fc/Fc+) pair with a redox potential believed to be 4.8 eV at the vacuum level, the electrochemical potentials of the HOMO energy levels were determined. The HOMO energy levels of the terpolymers were marginally lower than that of the control PM6 polymer (‒5.49 eV), which were ‒5.50, ‒5.52, ‒5.55, ‒5.56, and ‒5.57 eV for PTF3, PTF5, PTF10, PTF20, and PTF50, respectively (Figure 2e). The HOMO energy levels of the terpolymers effectively decreased due to the increased electronegativity^[^ ^21^ ^]^ of the TFB unit or steric hindrance effect caused by the amplification of its bulkiness.^[^ ^35^ ^]^ These findings suggest that by controlling the concentration of the TFB unit, the HOMO levels can be adjusted, and should thus be advantageous to attain a higher *V*_OC_ and reduced energy loss in OSCs.

**Figure 3:**
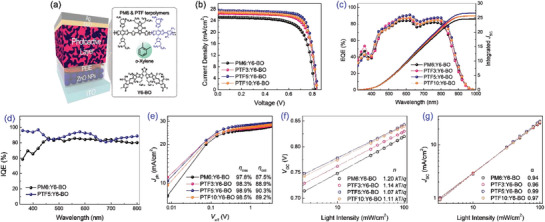
a) Device architecture and photoactive materials processed from *o*‐xylene. b) Best *J*‒*V* curves, c) EQE spectra with integrated *J*_SC_, d) IQE spectra and e) *J*_ph_‒*V*_eff_ characteristics of OSCs based on polymer donor:Y6‐BO. Dependence of f) *V*_OC_ and g) *J*_SC_ on the incident light intensity for polymer donor:Y6‐BO‐based devices.

The OSCs were fabricated by employing inverted device architecture, an ITO/ZnO NPs/PEIE/photoactive layer (polymer donor:Y6‐BO acceptor = 1.0:1.2)/MoO_X_/Ag to assess the photovoltaic properties (**Figure** **3**a). Table S1 and Figure S3, Supporting Information, present a comprehensive analysis of the optimization procedure for polymer donor:Y6‐BO at various weight ratios, offering detailed insights into the process. The best current density–voltage (*J*–*V*) curves are presented in Figure 3b and the comprehensive photovoltaic parameters are outlined in **Table** **2**.

**Table 2:** Detailed photovoltaic parameters of OSCs based on polymer donor:Y6‐BO processed from *o*‐xylene under an illumination of AM 1.5G at 100 mW cm^‒2^

Photoactive layer^a)^	*V*_OC_ [V]	*J*_SC_ [mA cm^‒2^]	Calc. *J*_SC_ [mA cm^‒2^]^b)^	FF [%]	PCE [%]	*µ*_h_ [cm^2^ V^‒1^ s^‒1^]^c)^	*µ*_e_ [cm^2^ V^‒1^ s^‒1^]^d)^	*µ*_h_/*µ*_e_
PM6:Y6‐BO	0.82 (0.81 ± 0.01)	25.2 (24.9 ± 0.31)	24.6	71.3 (70.1 ± 1.19)	15.1 (14.7 ± 0.42)	3.80 × 10^‒4^	2.63 × 10^‒4^	1.44
PTF3:Y6‐BO	0.83 (0.83 ± 0.01)	26.5 (26.3 ± 0.25)	25.3	74.0 (72.9 ± 1.12)	16.3 (16.0 ± 0.36)	4.63 × 10^‒4^	3.52 × 10^‒4^	1.32
PTF5:Y6‐BO	0.84 (0.84 ± 0.01)	28.0 (27.8 ± 0.22)	26.7	77.2 (76.4 ± 0.87)	18.2 (17.8 ± 0.34)	6.96 × 10^‒4^	6.25 × 10^‒4^	1.11
PTF10:Y6‐BO	0.84 (0.85 ± 0.01)	27.0 (26.7 ± 0.29)	25.7	75.1 (74.1 ± 1.07)	17.0 (16.8 ± 0.35)	5.09 × 10^‒4^	4.16 × 10^‒4^	1.22

^a)^ Inverted device architecture is ITO/ZnO NPs/PEIE/photoactive layer (*d* ≈ 130 nm)/MoO_X_/Ag. Photoactive materials were dissolved in *o*‐xylene and blend films were annealed at 130 °C for 10 min;

^b)^ *J*_SC_ values calculated from EQE spectra;

^c)^ Hole‐only device is ITO/PEDOT:PSS/photoactive layer (*d* ≈ 130 nm)/Au;

^d)^ Electron‐only device is ITO/ZnO NPs/PEIE/Photoactive layer (*d* ≈ 130 nm)/Ca/Al; The values in parentheses are average photovoltaic properties obtained from over 15 devices.

As demonstrated, the device based on PM6:Y6‐BO obtained a *V*_OC_ of 0.82 V, *J*_SC_ of 25.8 mA cm^−2^, and FF of 71.3%, leading to a PCE of 15.1%. Ultimately, the optimized device utilizing PTF5:Y6‐BO showed an outstanding PCE of 18.2%, with a *V*_OC_ of 0.84 V, *J*_SC_ of 28.0 mA cm^−2^, and FF of 77.2%. As anticipated, the *V*_OC_ increased with the rise in the proportion of CF3 in the terpolymer donor, which closely correlates with the lowering of the HOMO energy level of the polymer donors. Specifically, when the CF3 content increases from 0% to 50%, the corresponding *V*_OC_ value rises from 0.82 V to 0.88 V, respectively. The best *J*–*V* characteristics of the OSCs based on PTF20 and PTF50 can be found in Figure S4, Supporting Information, and the relevant photovoltaic parameters are tabulated in Table S2, Supporting Information. The improved photovoltaic performance observed in the PTF5‐based device is attributed to the simultaneous increase in all photovoltaic parameters of the *V*_OC_, *J*_SC_, and FF value. These photovoltaic results suggest that incorporating TFB as a third component for random polymerization is a viable approach to fine‐tune the electronic energy levels and improve photovoltaic performance.

The external quantum efficiency (EQE) spectra with integrated *J*_SC_ of the optimized devices are depicted in Figure 3c, exhibiting broad and high photo‐response characteristics. The calculated integrated *J*_SC_ values align well with those obtained from the *J*–*V* curves, with a discrepancy of less than 5%. The PTF5‐based device particularly exhibits an efficient photo‐to‐electron conversion among the other OSCs. The conversion of photons to electrons in OSCs depends on the exciton dissociation and charge extraction processes, which are responsible for converting photogenerated excitons into free charges and subsequently collected by respective electrodes. To investigate this process, the photogenerated current density (*J*_ph_) was plotted against the effective voltage (*V*_eff_) of the OSCs (Figure 3e). The evaluation of internal quantum efficiency (IQE) was carried out for the PM6 and PTF5‐based inverted OSCs with a thickness of 130 nm (Figure 3d). Remarkably, the PTF5‐based device exhibits an exceptional IQE value exceeding 80% across the wavelength range of 350–800 nm, surpassing that of PM6:Y6‐BO OSC. This significant increase in IQE strongly indicates that a substantial portion of absorbed photons efficiently generate charge carriers, which are then effectively collected at the electrodes. As a result, this enhanced charge carrier generation, transportation and collection contribute to improved photovoltaic performance. *J*_ph_ is defined as the difference between the light current density and the dark current density (*J*_L_ − *J*_D_). *V*_eff_ is calculated using the equation *V*_eff_ = *V*_0_ − *V*_a_, where *V*_0_ is the voltage at which *J*_ph_ = 0 and *V*_a_ is the applied bias voltage.^[^ ^36^ ^]^ Additionally, the exciton dissociation (*η*_diss_) and charge collection (*η*_coll_) can be determined according to formulas *η*_diss_ = *J*_ph_/*J*_sat_ and *η*_coll_ = *J*_max_/*J*_sat_, where *J*_sat_ is the saturated current density and *J*_max_ is the maximum current density. The OSCs based on PM6, PTF3, PTF5, and PTF10 exhibited an *η*_diss_ of approximately 97.8%, 98.3%, 98.9%, and 98.5%, respectively. Similarly, *η*_coll_ values were estimated to be 87.5%, 88.9%, 90.3%, and 89.2% for PM6, PTF3, PTF5, and PTF10‐based devices, respectively. These results suggest that all OSCs demonstrated effective exciton dissociation and charge collection properties. Notably, the PTF5‐based device exhibited the most efficient exciton dissociation and successful charge collections, which could account for its higher *J*_SC_ and FF values compared to the other devices.

To gain insight into the charge recombination mechanism in these devices, we investigated the dependence of *V*_OC_ and *J*_SC_ on the incident light intensity (*P*_light_), as shown in Figure 3f,g.^[^ ^37^ ^]^ Generally, the *V*_OC_–ln(*P*_light_) plot can provide information about the charge recombination property of OSCs under open‐circuit conditions. If the slope is closer to 1.0 *kT*/*q* (*k*, *T*, and *q* denote the Boltzmann constant, Kelvin temperature, and the elementary charge, respectively), the dominant loss mechanism is a bimolecular recombination. Conversely, a slope value of 2 *kT*/*q* implies that the devices are dominated by the trap‐assisted recombination.^[^ ^38^, ^39^ ^]^ Figure 3f shows that the slopes for PM6, PTF3, PTF5 and PTF10‐based OSCs are 1.20 *kT*/*q*, 1.14 *kT*/*q*, 1.07 *kT*/*q*, and 1.11 *kT*/*q*, respectively. These values suggest that the PTF5‐based device has the lowest trap‐assisted charge recombination in the bulk.

The charge recombination status in these OSCs was also investigated via the dependence of *J*_SC_ on the incident light intensity (Figure 3g). The power law equation *J*_SC_ ∝ (*P*_light_)^*α*^ is used to describe the correlation between *J*_SC_ and *P*_light_, where *α* is an exponential factor. A weak bimolecular recombination is indicated by an *α* value close to 1. The *α* values for the OSCs with different polymer donors range from 0.94 to 0.99. Among the devices, PTF5‐based OSC showed effectively suppressed bimolecular recombination; thus, the addition of 5% TFB into PM6 polymer backbone benefits from the reduced trap‐assisted and bimolecular recombination, leading to improved photovoltaic OSC performance.

The hole and electron transport properties of the OSCs based on various polymer donors were measured via the space‐charge limited current (SCLC) method. The dark *J*‒*V* curves of the hole‐only and electron‐only devices were plotted, as shown in Figure S5, Supporting Information, and the calculated hole (*µ*_h_) and electron (*µ*_e_) mobility are listed in Table 2. The *µ*_h_ for PM6, PTF3, PTF5, and PTF10‐based OSCs was estimated to be 3.80 × 10^−4^, 4.63 × 10^−4^, 6.96 × 10^−4^, and 5.09 × 10^−4^ cm^2^ V^−1^ s^−1^, respectively. The corresponding *µ*_e_ was 2.63 × 10^−4^, 3.52 × 10^−4^, 6.25 × 10^−4^, and 4.16 × 10^−4^ cm^2^ V^−1^ s^−1^, respectively. Compared to the other devices, the PTF5‐based device demonstrates high and balanced charge transport, which may help reduce charge recombination. It likely that the BHJ morphology based on PTF5 improves interpenetrating networks, thus contributing to a higher FF of 77.2%.

**Figure 4:**
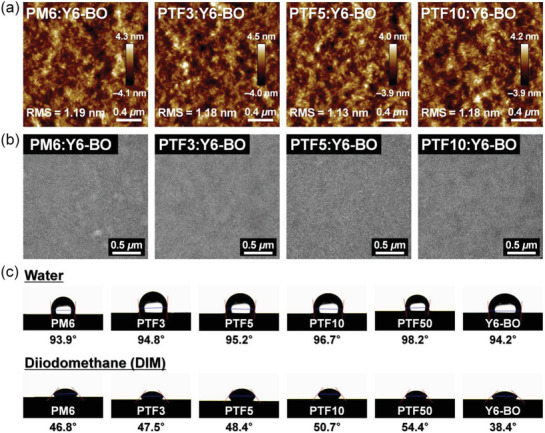
a) AFM height images and b) TEM images for polymer donor:Y6‐BO blend films. c) Contact angles of PM6, terpolymers, and Y6‐BO to water and diiodomethane.

The surface and bulk morphology of photoactive films were investigated and their atomic force microscopy (AFM) and transmission electron microscopy (TEM) images are shown in **Figure** **4** and Figures S6 and S7, Supporting Information. As the content of TFB increases in the PM6 polymer backbone, there is a gradual decrease in the root mean square (RMS) values. The PTF5‐based film exhibits the smallest RMS value of 1.13 nm among the photoactive films, which is ascribed to the most homogeneously distributed morphology formed therein (Figure 4a; Figure S6, Supporting Information). As is known, the smooth surface of the photoactive layer can lead to a reduced trap recombination and improved interfacial contact, which is beneficial for achieving a higher *J*_SC_ and FF.^[^ ^40^ ^]^ The TEM images (Figure 4b; Figure S7, Supporting Information) also reveal that the PTF5:Y6‐BO photoactive materials are segregated into nanoscale domains with discernible fibrous structures, which may facilitate efficient charge transport.

To determine the miscibility and aggregation properties between the polymer donor and Y6‐BO acceptor, the contact angle measurements of water and diiodomethane were used to calculate the surface tension, as shown in Figure 4c, and the relevant parameters are summarized in Table S3, Supporting Information. The Flory–Huggins interaction parameter (*χ*) is a crucial index to quantify the molecular interactions between these photoactive constituents. In general, the relatively small *χ* value indicates strong attractive interactions between the two components in the mixtures, while the high *χ* value leads to the immiscibility of two phases in the blend films.^[^ ^41^ ^]^ The *χ* of the donor–acceptor values for PM6/Y6‐BO, PTF3/Y6‐BO, PTF5/Y6‐BO, PTF10/Y6‐BO, and PTF50/Y6‐BO were 0.12, 0.16, 0.18, 0.29, and 0.45, respectively. Thus, as the proportion of the CF3 component increased in the PM6 polymer backbone, its *χ* values increased, indicating a decrease in miscibility and an increase in phase aggregation. Due to the optimum *χ* value of the binary PTF5/Y6‐BO system, the incorporation of 5% TFB into the PM6 polymer backbone could not only induce suitable phase separation with interpenetrating fibrous network structures but also reduce bimolecular recombination, helping to explain the higher device FF value.

Grazing incidence wide angle X‐ray scattering (GIWAXS) measurements were performed to analyze the molecular packing and orientation of neat polymer films depending on the insertion ratio of the TFB unit. The 2D GIWAXS pattern images and line‐cut profiles of each neat film processed in *o*‐xylene are presented in Figure S8, Supporting Information, and the corresponding detailed crystallographic parameters are provided in Figure S9 and Table S4, Supporting Information.

As shown in Figure S8a, Supporting Information, compared to PM6, the neat films except for PTF50 exhibited similar scattering patterns, indicating no significant difference in molecular packing behavior in PTF3, PTF5, and PTF10. However, unlike other neat films, distinct peaks at *q*_z_ ≈ 0.91 Å^‒1^ and 1.21 Å^‒1^ did not appear in PTF50 (Figure S8c, Supporting Information), which can be regarded as a complete change in the packing structure. It was confirmed that the scattering peaks located at *q*_z_ ≈ 0.91 Å^‒1^ and 1.21 Å^‒1^ were (300) and (400), respectively, according to the calculated *d*‐spacing values (Table S5, Supporting Information). The paracrystalline disorder parameter (*g*), defined as the statistical standard deviation from its average lattice position, was estimated by fitting the Hosemann plot (*δb*‒*h* ^2^) from (*h*00) diffraction peaks (Figure S10, Supporting Information), where *δb* is the reciprocal value of the coherence length (*L*_C_), and *h* is the diffraction peak order.^[^ ^42^, ^43^, ^44^ ^]^ The calculated *g* values of PM6, PTF3, PTF5, and PTF10 were found to be 5.68%, 5.71%, 5.20%, and 5.53%, respectively. In comparison with PM6, PTF3 showed a similar *g* value, whereas PTF5 and PTF10 exhibited lower *g* values, indicating that TFB insertion helped the terpolymer form well‐ordered crystalline structures. In addition, the introduction of TFB demonstrated a decrease in the lamellar stacking distance (*d*_(100)_) and an increase in coherence length (*L*_C(100)_) along the IP direction (Figure S9 and Table S4, Supporting Information), suggesting that the terpolymers tend to have face‐on dominant molecular orientations compared to PM6.

The above phenomena were identified by extracting azimuthal profiles from the (010) scattering peaks of each neat film (Figure S11 and Table S6, Supporting Information). As shown in the scattering pattern images (Figure S8a, Supporting Information) and azimuthal cut plots (Figure S11a, Supporting Information), the neat films from PM6 to PTF10 have a bimodal lamellar packing structure, while PTF50 has a face‐on dominant molecular orientation. Meanwhile, compared to PM6, the degree of face‐on orientation tended to increase along with the insertion ratio of TFB (Figure S11b, Supporting Information). This increase could be due to intermolecular aggregation and non‐covalent interactions facilitated by the CF3 group, which are known to promote efficient charge transport in materials. However, even though PTF50 exhibited the most predominant face‐on orientation, the distinct trends of decreasing *L*_C_ and increasing *π*‒*π* stacking distance (*d*_(010)_) compared to other neat films (Figure S9, Supporting Information) suggest that inferior intermolecular packing hinders charge transport.

**Figure 5:**
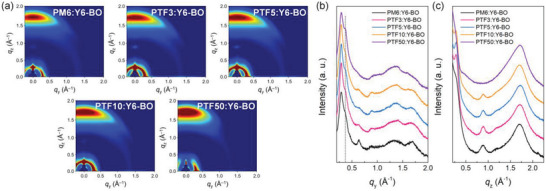
a) 2D GIWAXS scattering pattern images of polymer donor:Y6‐BO blend films. Line‐cut profiles for b) in‐plane (IP) direction and c) out‐of‐plane (OOP) directions, respectively.

To further understand the characteristics of the BHJ morphology according to the TFB insertion ratio in PM6, GIWAXS measurements of photoactive layers for polymer donor:Y6‐BO were conducted. The GIWAXS scattering pattern images and the corresponding line‐cut profiles for the blend films are presented in **Figure** **5**, and the detailed molecular packing parameters can be found in Table S7, Supporting Information.

As shown in Figure 5a, all blend films exhibited a face‐on dominant packing orientation. In the same manner as the neat films, the blend films from the PM6:Y6‐BO to PTF10:Y6‐BO showed similar BHJ morphology, while the PTF50:Y6‐BO blend film exhibited significantly different molecular packing behavior. In addition, as the proportion of TFB increased, the characteristic peak of Y6‐BO located at *q*_y_ ≈ 0.37 Å^‒1^ gradually became distinct, indicating that the miscibility between the polymer donor and Y6‐BO reduced (Figure 5b). The molecular miscibility according to the insertion ratio of TFB is consistent with the *χ* values calculated from contact angle measurements (Figure 4c; Table S3, Supporting Information). Compared to the PM6:Y6‐BO blend films, the *L*_C_ value of *π*‒*π* stacking (*L*_C(010)_) for terpolymer‐based blend films decreased, while the *L*_C_ value of lamellar ordering (*L*_C(100)_) for terpolymer‐based blend films increased (Table S7, Supporting Information). The variation of *L*_C_ values demonstrates that the incorporation of TFB into PM6 can effectively regulate the molecular packing and orientation of photoactive materials.

Similar to pristine polymer films, a reduction in *d*_(010)_ along the OOP direction is observed in the blend films. The *d*_(010)_ value decreased from 3.68 Å for PM6:Y6‐BO to 3.65 Å for PTF5:Y6‐BO, which likely increased the intermolecular charge hopping efficiency in PTF5‐based OSCs. Above all, PTF5:Y6‐BO photoactive film exhibited a desirable crystalline morphology owing to its well‐controlled molecular aggregation and miscibility, which resulted in a reduction in charge recombination and an increase in the FF value. This finding is consistent with the results obtained from AFM, TEM, and contact angle measurements (Figure 4).

**Figure 6:**
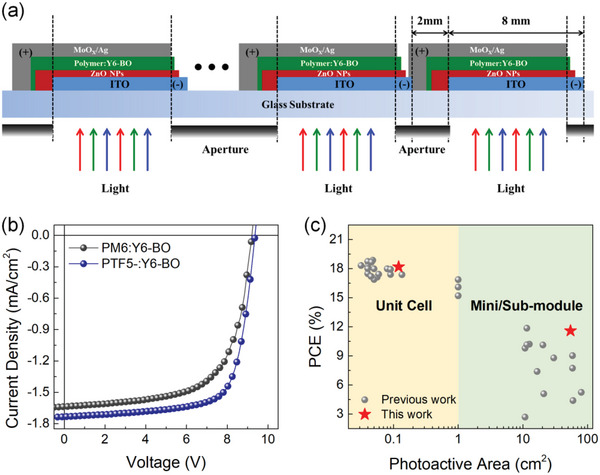
a) Schematic device illustration of the large‐area OSC. b) Best *J*‒*V* characteristics large‐area OSCs based on PM6:Y6‐BO and PTF5:Y6‐BO. c) Plot of photoactive area versus PCE for small and large‐area OSCs in the binary system.

More encouragingly, the PTF5:Y6‐BO photoactive system was selected to demonstrate highly efficient large‐area OSCs. These sub‐module devices were fabricated using a D‐bar coater in air (Figure S12, Supporting Information). The sub‐module fabrication design is illustrated in **Figure** **6**a and the corresponding best *J*‒*V* curves are shown in Figure 6b. The champion large‐area OSC based on the PTF5:Y6‐BO system, which was fabricated using a halogen‐free solvent *o*‐xylene, achieved an excellent PCE of 11.6% with a certified PCE of 11.5% (Figure S13, Supporting Information). This sub‐module device with an interconnection of up to eleven subcells in series (a total photoactive area of 54.45 cm^2^) exhibits a *V*_OC_ of 9.32 V, *J*_SC_ of 1.73 mA cm^−2^, and FF of 71.9%. Conversely, the control PM6‐based sub‐module showed a poor PCE of 8.73% with a *V*_OC_ of 9.12 V, *J*_SC_ of 1.48 mA cm^−2^, and FF of 64.7% (**Table** **3**). This improvement in PTF5‐based sub‐module performance may be attributed to the uniform and pinhole‐free photoactive film, leading to minimized cell‐to‐module loss (Figure S14, Supporting Information). To the best of our knowledge, these PCEs are the highest value among small and large‐area OSCs with a binary system (Figure 6c; Table S8, Supporting Information).

**Table 3:** Detailed photovoltaic parameters of large‐area OSCs based on PM6:Y6‐BO and PTF5:Y6‐BO photoactive layer processed from *o*‐xylene under an illumination of AM 1.5G at 100 mW cm^‒2^ (Aperture size = 54.45 cm^2^)

Photoactive layer^a)^	*V*_OC_ [V]	*J*_SC_ [mA cm^‒2^]	FF [%]	PCE [%]	PCE^large^/PCE^small^
PM6:Y6‐BO	9.12 (9.02 ± 0.10)	1.48 (1.45 ± 0.04)	64.7 (60.4 ± 4.60)	8.73 (7.08 ± 1.73)	0.58
PTF5:Y6‐BO	9.32 (9.23 ± 0.08)	1.73 (1.71 ± 0.02)	71.9 (69.4 ± 2.63)	11.6 (10.2 ± 1.18)	0.64

^a)^ Inverted device architecture is ITO/ZnO NPs/Photoactive layer (*d* ≈ 130 nm)/MoO_X_/Ag. Photoactive materials were dissolved in *o*‐xylene and blend films were annealed at 130 °C for 10 min; The values in parentheses are average photovoltaic properties obtained from over 5 devices.

## 3 Conclusion

In summary, a random copolymerization strategy to synthesize a series of high‐performance D‐A1‐D‐A2 type terpolymers (PTF3, PTF5, PTF10, PTF20, and PTF50) was developed by adding an electron‐withdrawing TFB unit to the PM6 polymer backbone. Owing to the lower HOMO energy level, optimal molecular packing, and more desirable aggregation/miscibility morphology by incorporation of the TFB, the best binary OSC based on PTF5:Y6‐BO processed from halogen‐free solvent *o*‐xylene achieved an impressive PCE of 18.2 % with an enhanced *V*_OC_ of 0.84 V, *J*_SC_ of 28.0 mA cm^−2^, and FF of 77.2% relative to that of the PM6‐based device (PCE = 15.1%, *V*_OC_ = 0.82 V, *J*_SC_ = 25.8 mA cm^−2^, and FF = 71.3%). Furthermore, we observed that a bar‐coated sub‐module device based on PTF5:Y6‐BO with a aperture size of 54.45 cm^2^ demonstrated a remarkable PCE of 11.6% (certified PCE of 11.5%). To the best of our knowledge, this unit and sub‐module PCE is one of the highest values reported to date in binary OSCs, even processed from halogen‐free solvent. This study demonstrates that random copolymerization with trifluoromethyl substitution is a feasible and effective approach to fine‐tune photoactive morphology and thereby enhance device performance.

## Conflict of Interest

The authors declare no conflict of interest.

## Supporting information

Supporting Information

Supplemental Table 1

Supplemental Table 2

Supplemental Table 3

## Data Availability

The data that support the findings of this study are available on request from the corresponding author. The data are not publicly available due to privacy or ethical restrictions.

## References

[1] K. Leo , Nat. Rev. Mater. 2016, 1, 16056.

[2] D. Hu , Q. Yang , H. Chen , F. Wobben , V. M. Le Corre , R. Singh , T. Liu , R. Ma , H. Tang , L. J. A. Koster , T. Duan , H. Yan , Z. Kan , Z. Xiao , S. Lu , Energy Environ. Sci. 2020, 13, 2134.

[3] C. Sun , F. Pan , H. Bin , J. Zhang , L. Xue , B. Qiu , Z. Wei , Z. G. Zhang , Y. Li , Nat. Commun. 2018, 9, 743. 29467393 10.1038/s41467-018-03207-x PMC5821836

[4] S. B. Darling , F. You , RSC Adv. 2013, 3, 17633.

[5] Z. Abbas , J. Shin , R. Atla , S. Rasool , C. E. Song , H. K. Lee , S. K. Lee , W. S. Shin , W. W. So , S. K. Kwon , Y. H. Kim , J. C. Lee , ACS Appl. Mater. Interfaces 2018, 10, 39107. 30350940 10.1021/acsami.8b14888

[6] Z. Abbas , S. U. Ryu , M. Haris , C. E. Song , H. K. Lee , S. K. Lee , W. S. Shin , T. Park , J.‐C. Lee , Nano Energy 2022, 101, 107574.

[7] J. Song , L. Zhu , C. Li , J. Xu , H. Wu , X. Zhang , Y. Zhang , Z. Tang , F. Liu , Y. Sun , Matter 2021, 4, 2542.

[8] H. Tang , H. Chen , C. Yan , J. Huang , P. W. K. Fong , J. Lv , D. Hu , R. Singh , M. Kumar , Z. Xiao , Z. Kan , S. Lu , G. Li , Adv. Energy Mater. 2020, 10, 2001076.

[9] H. Wang , H. Lu , Y. N. Chen , G. Ran , A. Zhang , D. Li , N. Yu , Z. Zhang , Y. Liu , X. Xu , W. Zhang , Q. Bao , Z. Tang , Z. Bo , Adv. Mater. 2022, 34, 2105483. 10.1002/adma.202105483 34773717

[10] H. Ning , Q. Jiang , P. Han , M. Lin , G. Zhang , J. Chen , H. Chen , S. Zeng , J. Gao , J. Liu , F. He , Q. Wu , Energy Environ. Sci. 2021, 14, 5919.

[11] X. Dong , D. Hu , P. Chen , X. Dai , C. Hu , Z. Xiao , S. Lu , J. Semicond. 2020, 41, 122201.

[12] L. Zhou , L. Meng , J. Zhang , C. Zhu , S. Qin , I. Angunawela , Y. Wan , H. Ade , Y. Li , Adv. Funct. Mater. 2022, 32, 2109271.

[13] Y. Cui , Y. Xu , H. Yao , P. Bi , L. Hong , J. Zhang , Y. Zu , T. Zhang , J. Qin , J. Ren , Z. Chen , C. He , X. Hao , Z. Wei , J. Hou , Adv. Mater. 2021, 33, 2102420.

[14] J. Yuan , Y. Zhang , L. Zhou , G. Zhang , H.‐L. Yip , T.‐K. Lau , X. Lu , C. Zhu , H. Peng , P. A. Johnson , M. Leclerc , Y. Cao , J. Ulanski , Y. Li , Y. Zou , Joule 2019, 3, 1140.

[15] Z. Zhou , W. Liu , G. Zhou , M. Zhang , D. Qian , J. Zhang , S. Chen , S. Xu , C. Yang , F. Gao , H. Zhu , F. Liu , X. Zhu , Adv. Mater. 2020, 32, 1906324. 10.1002/adma.201906324 31815332

[16] S. Li , C.‐Z. Li , M. Shi , H. Chen , ACS Energy Lett. 2020, 5, 1554.

[17] J. Zhang , W. Liu , G. Zhou , Y. Yi , S. Xu , F. Liu , H. Zhu , X. Zhu , Adv. Energy Mater. 2020, 10, 1903298.

[18] J. Wang , S. Wang , C. Duan , F. J. M. Colberts , J. Mai , X. Liu , X. e. Jia , X. Lu , R. A. J. Janssen , F. Huang , Y. Cao , Adv. Energy Mater. 2017, 7, 1702033.

[19] M. Zhang , X. Guo , W. Ma , H. Ade , J. Hou , Adv. Mater. 2015, 27, 4655. 26173152 10.1002/adma.201502110

[20] Y. Cui , H. Yao , L. Hong , T. Zhang , Y. Xu , K. Xian , B. Gao , J. Qin , J. Zhang , Z. Wei , J. Hou , Adv. Mater. 2019, 31, 1808356. 10.1002/adma.201808356 30779391

[21] J. Wu , G. Li , J. Fang , X. Guo , L. Zhu , B. Guo , Y. Wang , G. Zhang , L. Arunagiri , F. Liu , H. Yan , M. Zhang , Y. Li , Nat. Commun. 2020, 11, 4612. 32929082 10.1038/s41467-020-18378-9 PMC7490407

[22] X. Guo , Q. Fan , J. Wu , G. Li , Z. Peng , W. Su , J. Lin , L. Hou , Y. Qin , H. Ade , L. Ye , M. Zhang , Y. Li , Angew. Chem., Int. Ed. 2021, 60, 2322. 10.1002/anie.202010596 33058442

[23] H. Sun , T. Liu , J. Yu , T.‐K. Lau , G. Zhang , Y. Zhang , M. Su , Y. Tang , R. Ma , B. Liu , J. Liang , K. Feng , X. Lu , X. Guo , F. Gao , H. Yan , Energy Environ. Sci. 2019, 12, 3328.

[24] J. Shao , C. Liao , X. Xu , M. Deng , L. Yu , R. Li , Q. Peng , Chem. Mater. 2022, 34, 7971.

[25] Y. Zafrani , G. Sod‐Moriah , D. Yeffet , A. Berliner , D. Amir , D. Marciano , S. Elias , S. Katalan , N. Ashkenazi , M. Madmon , E. Gershonov , S. Saphier , J. Med. Chem. 2019, 62, 5628. 31091098 10.1021/acs.jmedchem.9b00604

[26] H. Lai , Q. Zhao , Z. Chen , H. Chen , P. Chao , Y. Zhu , Y. Lang , N. Zhen , D. Mo , Y. Zhang , F. He , Joule 2020, 4, 688.

[27] C. Yao , Y. Zhu , K. Gu , J. Zhao , J. Ning , D. F. Perepichka , Y.‐L. Loo , H. Meng , J. Mater. Chem. A 2020, 8, 12149.

[28] L. Lu , L. Yu , Adv. Mater. 2014, 26, 4413. 24677495 10.1002/adma.201400384

[29] J. Liang , M. Pan , G. Chai , Z. Peng , J. Zhang , S. Luo , Q. Han , Y. Chen , A. Shang , F. Bai , Y. Xu , H. Yu , J. Y. L. Lai , Q. Chen , M. Zhang , H. Ade , H. Yan , Adv. Mater. 2020, 32, 2003500. 10.1002/adma.202003500 33185952

[30] N. Yin , L. Wang , Y. Lin , J. Yi , L. Yan , J. Dou , H. B. Yang , X. Zhao , C.‐Q. Ma , Beilstein J. Org. Chem. 2016, 12, 1788. 27829886 10.3762/bjoc.12.169 PMC5082721

[31] J. Wu , X. Guo , M. Xiong , X. Xia , Q. Li , J. Fang , X. Yan , Q. Liu , X. Lu , E. Wang , D. Yu , M. Zhang , Chem. Eng. J. 2022, 446, 137424.

[32] J. Kim , M. Kyeong , J.‐W. Ha , H. Ahn , J. Jung , S. Seo , T. N.‐L. Phan , C. Lee , S. C. Yoon , B. J. Kim , S.‐J. Ko , J. Mater. Chem. A 2021, 9, 27551.

[33] Y. Lei , P. Deng , J. Li , M. Lin , F. Zhu , T. W. Ng , C. S. Lee , B. S. Ong , Sci. Rep. 2016, 6, 24476. 27091315 10.1038/srep24476 PMC4835732

[34] G.‐J. N. Wang , F. Molina‐Lopez , H. Zhang , J. Xu , H.‐C. Wu , J. Lopez , L. Shaw , J. Mun , Q. Zhang , S. Wang , A. Ehrlich , Z. Bao , Macromolecules 2018, 51, 4976.

[35] T. Wang , R. Sun , M. Shi , F. Pan , Z. Hu , F. Huang , Y. Li , J. Min , Adv. Energy Mater. 2020, 10, 2000590.

[36] S. R. Cowan , A. Roy , A. J. Heeger , Phys. Rev. B 2010, 82, 245207.

[37] L. J. A. Koster , V. Mihailetchi , H. Xie , P. W. Blom , Appl. Phys. Lett. 2005, 87, 203502.

[38] S. Xu , L. Feng , J. Yuan , Z. G. Zhang , Y. Li , H. Peng , Y. Zou , ACS Appl. Mater. Interfaces 2017, 9, 18816. 28530392 10.1021/acsami.7b03947

[39] J. Vollbrecht , V. V. Brus , Org. Electron. 2020, 86, 105905.

[40] L. Nian , K. Gao , Y. Jiang , Q. Rong , X. Hu , D. Yuan , F. Liu , X. Peng , T. P. Russell , G. Zhou , Adv. Mater. 2017, 29, 1700616. 10.1002/adma.201700616 28589656

[41] S. Nilsson , A. Bernasik , A. Budkowski , E. Moons , Macromolecules 2007, 40, 8291.

[42] D. Yoo , B. Nketia‐Yawson , S.‐J. Kang , H. Ahn , T. J. Shin , Y.‐Y. Noh , C. Yang , Adv. Funct. Mater. 2015, 25, 586.

[43] M. Kim , W. T. Park , S. A. Park , C. W. Park , S. U. Ryu , D. H. Lee , Y. Y. Noh , T. Park , Adv. Funct. Mater. 2019, 29, 1805994.

[44] S. U. Ryu , Z. Abbas , A. Cho , H. Lee , C. E. Song , H. K. Lee , S. K. Lee , W. S. Shin , S. J. Moon , T. Park , H. I. Kim , J. C. Lee , Adv. Energy Mater. 2020, 10, 1903846.

