# Supplementary material for: Trifluoromethyl‐Substituted Conjugated Random Terpolymers Enable High‐Performance Small and Large‐Area Organic Solar Cells Using Halogen‐Free Solvent
Source: Adv Sci (Weinh). 2023 Jun 25;10(24):2302376. doi: 10.1002/advs.202302376 (PMC10460891; doi:10.1002/advs.202302376)
Supplement: Supplementary file 1 — Supporting Information [file ADVS-10-2302376-s002.pdf]

ADVANCED SCIENCE
Open Access

Supporting Information

for *Adv. Sci.*, DOI 10.1002/advs.202302376

Trifluoromethyl-Substituted Conjugated Random Terpolymers Enable High-Performance Small and Large-Area Organic Solar Cells Using Halogen-Free Solvent

Zia Ur Rehman, Muhammad Haris, Seung Un Ryu, Muhammad Jahankhan, Chang Eun Song, Hang Ken Lee, Sang Kyu Lee, Won Suk Shin, Taiho Park* and Jong-Cheol Lee**

Supporting Information

Trifluoromethyl-Substituted Conjugated Random Terpolymers Enable High-Performance Small and Large-Area Organic Solar Cells using Halogen-Free Solvent

Zia Ur Rehman†, Muhammad Haris†, Seung Un Ryu†, Muhammad Jahankhan, Chang Eun Song, Hang Ken Lee, Sang Kyu Lee, Won Suk Shin, Taiho Park*, and Jong-Cheol Lee**

Z. Rehman, M. Haris, M. Jahankhan, Dr. C. E. Song, Dr. H. K. Lee, Dr. S. K. Lee, Dr. W. S. Shin, Dr. J.-C. Lee,
Advanced Energy Materials Research Center, Korea Research Institute of Chemical Technology (KRICT), Daejeon, 34114, Republic of Korea
Email: songce@krict.re.kr, leejc@krict.re.kr

Z. Rehman, M. Haris, M. Jahankhan, Prof. C. E. Song, Prof. S. K. Lee, Prof. W. S. Shin, Prof. J.-C. Lee,
Advanced Materials and Chemical Engineering, University of Science and Technology (UST), Daejeon, 34113, Republic of Korea

S. U. Ryu, Prof. T. Park
Department of Chemical Engineering, Pohang University of Science and Technology (POSTECH), Pohang, Gyeongsangbuk-do 37673, Republic of Korea
Email: taihopark@postech.ac.kr

†First author
Z. U. Rehman, M. Haris, and S. U. Ryu contributed equally to this work.

*Corresponding author
E-mail address: songce@krict.re.kr (C. E. Song), taihopark@postech.ac.kr (T. Park), and leejc@krict.re.kr (J.-C. Lee)

Contents

Experimental details

1. **Synthetic procedure details**
2. **Measurements**
3. **Device fabrication**
4. **Photovoltaic characterization**
5. **Hole and electron only device and mobility measurements**
6. **Grazing-incidence wide-angle X-ray scattering (GIWAXS) analysis**

Figure S1. (a) Thermogravimetric analysis (TGA) traces and (b) Differential scanning calorimetry (DSC) thermograms of PM6 and terpolymers.
Figure S2. Cyclic voltammogram (CV) characteristics of PM6 and terpolymers.
Figure S3. *J-V* characteristics of polymer donor:Y6-BO OSCs with different weight ratios of (a) PM6, (b) PTF3, (b) PTF5, and (b) PTF10 under AM 1.5G illumination.
Figure S4. Best *J–V* characteristic curves of PTF20:Y6-BO and PTF50:Y6-BO-based OSCs.
Figure S5. SCLC characteristic curves of (a) hole-only and (b) electron-only devices based on donor polymer:Y6-BO photoactive layers.
Figure S6. AFM phase images of donor polymer:Y6-BO blend films.
Figure S7. High-magnification TFM images of donor polymer:Y6-BO blend films.
Figure S8. (a) 2D GIWAXS scattering pattern images of PM6 and terpolymers. Line-cut profiles for (b) in-plane (IP) direction and (c) out-of-plane (OOP) directions, respectively.
Figure S9. *d*-spacing and L_C values of neat films for (a) lamellar stacking and (b) π–π stacking along the IP direction. (c) *d*-spacing and L_C values of neat films for π–π stacking along the OOP direction.
Figure S10. Hosemann plots (δb–h^2) of PM6 and terpolymer neat films extracted from line-cut profiles for (*h*00) of GIWAXS in the OOP direction.
Figure S11. (a) Azimuthal cut plots of (010) scattering peaks for PM6 and terpolymer neat films. (b) Face-on and edge-on orientation distribution of each neat film.
Figure S12. Schematic illustration of the large-area OSC fabrication procedure using the D-bar coater.
Figure S13. *J–V* curve of the large-area OSC based on PTF5:Y6-BO photoactive layer certified by Korea Institute of Energy Research (KIER).

Figure S14. Images (a) after coating the PM6:Y6-BO photoactive material and (b) after metal deposition on the PM6:Y6-BO photoactive layer. Images (a) after coating the PTF5:Y6-BO photoactive material and (b) after metal deposition on the PTF5:Y6-BO photoactive layer.
Figure S15. ^{19}F NMR spectrum of PM6 polymer donor.
Figure S16. ^{19}F NMR spectrum of PTF3 polymer donor.
Figure S17. ^{19}F NMR spectrum of PTF5 polymer donor.
Figure S18. ^{19}F NMR spectrum of PTF10 polymer donor.
Figure S19. ^{19}F NMR spectrum of PTF20 polymer donor.
Figure S20. ^{19}F NMR spectrum of PTF50 polymer donor.

Table S1. Photovoltaic parameters of OSCs with different weight ratios of polymer donor:Y6-BO photoactive layer under an illumination of air mass 1.5 global (AM 1.5G), 100 mW/cm^2.
Table S2. Detailed photovoltaic parameters of OSCs based on PTF20:Y6-BO and PTF50:Y6-BO photoactive layers processed with *o*-xylene under an illumination of AM 1.5G at 100 mW cm^{-2}.
Table S3. Summary of contact angles for water and diiodomethane, surface energy, and Flory-Huggins interaction parameters of Y6-BO and donor polymers.
Table S4. Summary of crystallographic parameters for donor polymer and Y6-BO neat films.
Table S5. Summary of (*h*00) scattering peaks and the paracrystalline disorder parameter (g) for PM6 and terpolymers along the OOP direction.
Table S6. Orientation distribution of PM6 and terpolymer neat films extracted from (010) scattering peaks.
Table S7. Summary of crystallographic parameters for donor polymer:Y6-BO blend films.
Table S8. Summary of photoactive area and PCE of representative binary OSCs based on unit and mini/sub-module devices.

Experimental details

Materials

All reactions and manipulations were operated under N_2 and Ar atmosphere. All the starting materials were purchased from commercial suppliers and used without further purification. PM6 and Y6-BO were purchased from Brilliant Matters and used without further purification. All chemicals and reagents were used as received from commercial sources without further purification. (4,8-bis(5-(2-ethylhexyl)-4-fluorothiophen-2-yl)benzo[1,2-*b*:4,5-*b*']dithiophene-2,6-diyl)bis(trimethylstannane) (BDT2F-Sn) and 1,3-bis(5-bromothiophen-2-yl)-5,7-bis(2-ethylhexyl)benzo[1,2-*c*:4,5-*c*']dithiophene-4,8-dione (BDD-Br) were synthesized according to the previous reports.[1,2]

1. Synthetic procedure details

1) Synthesis of monomers

Synthesis of 1,3-bis(5-bromothiophen-2-yl)-5,7-bis(2-ethylhexyl)benzo[1,2-c:4,5-c']dithiophene-4,8-dione (BDD-Br)

To a solution of 1,3-bis(2-ethylhexyl)-5,7-di(thiophen-2-yl)benzo[1,2-*c*:4,5-*c*']dithiophene-4,8-dione (BDD) (2.7 g, 4.43 mmol) in DMF (50 ml), NBS (1.9 g, 10.63 mmol) was added in one portion. The reaction mixture was stirred at room temperature for 3 h. After that, the reaction mixture was quenched with water, extracted with $CHCl_3$. The organic layer was washed with brine and dried over anhydrous $MgSO_4$. After removing the solvent, the residue was purified by column chromatography on silica gel with hexane/methylene chloride (5:1) to give red solid (2.4 g, 71%). ^{1}H NMR (400 MHz, $CDCl_3$), δ (ppm): 7.39 (d, 2H), 7.03 (d, 2H), 3.27 (m, 4H), 1.74(m, 2H), 1.41−1.32 (m, 16H), 0.94−0.90 (m, 12H).

Synthesis of (4,8-bis(5-(2-ethylhexyl)-4-fluorothiophen-2-yl)benzo[1,2-b:4,5-b']dithiophene-2,6-diyl)bis(trimethylstannane) (BDT2F-Sn)

n-BuLi (5.1 ml, 1.6 M, 8.13 mmol) was added to a stirred solution of 4,8-bis(5-(2-ethylhexyl)-4-fluorothiophen-2-yl)benzo[1,2-*b*:4,5-*b*']dithiophene (BDT2F) (2.0 g, 3.25 mmol) in THF at –78 °C, and reaction mixture was stirred at this temperature for 1 h followed by the addition of trimethyltin chloride (8.1 ml, 1.0 M, 8.13 mmol) in THF at –78 °C. The reaction was then

quenched with water after 2 h stirring at room temperature and extracted with hexane. The organic layer was then washed with KF solution and dried over anhydrous $MgSO_4$. After removing the solvent, the residue was purified by recrystallization with ethanol to give off white crystalline solid (2.0 g, 67%). ^{1}H NMR (400 MHz, $CDCl_3$), δ (ppm): 7.67 (s, 2H), 7.16 (s, 2H), 2.79 (d, 4H), 1.68 (m, 2H), 1.48−1.30 (m, 16H), 0.94 (m, 12H), 0.42 (s, 18H).

2) Synthesis of polymers

General synthetic procedure of the polymers

BDT2F-Sn (100 mg, 0.1063 mmol) and BDD-Br and 1,4-dibromo-2,5-bis(trifluoromethyl) benzene (TFB-Br) monomers at varied ratios of 100/0, 0.97/0.03, 0.95/0.05, 0.90/0.10, 0.80/0.20, 0.50/0.50 were taken in a microwave vial followed by the addition of $Pd(PPh_3)_4$ (4.9 mg, 4%) in glovebox. After adding toluene (3 ml), the solutions were flushed with argon 10 min and reactions were stirred at reflux for 24 h. The reaction mixture was cooled down to room temperature and added to 100 ml of methanol. The precipitated solid was collected by filtration and subjected to Soxhlet extraction with methanol, acetone, hexane, methylene chloride, methylene chloride/chloroform and finally with chloroform. Subsequently, the solid was again precipitated in methanol, filtered and dried under vacuum to get polymers with yields around 75%.

PM6: BDT2F-Sn (100 mg, 0.1063 mmol), BDD-Br (81.5 mg, 0.1063 mmol). (110 mg). M_n = 159 kDa, PDI = 3.51.

PTF3: BDT2F-Sn (100 mg, 0.1063 mmol), BDD-Br (79.0 mg, 0.1031 mmol), TFB-Br (1.2 mg, 0.0032 mmol). (117 mg). M_n = 168 kDa, PDI = 4.38

PTF5: BDT2F-Sn (100 mg, 0.1063 mmol), BDD-Br (77.4 mg, 0.1010 mmol), TFB-Br (2.0 mg, 0.0053 mmol). (115 mg). M_n = 158 kDa, PDI = 4.04.

PTF10: BDT2F-Sn (100mg, 0.1063mmol), BDD-Br (73.4mg, 0.0957mmol), TFB-Br (3.9 mg, 0.0106 mmol). (116 mg). M_n = 148 kDa, PDI = 5.00.

PTF20: BDT2F-Sn (100 mg, 0.1063 mmol), BDD-Br (65.2 mg, 0.0850 mmol), TFB-Br (7.9 mg, 0.0213 mmol). (110 mg). M_n = 140 kDa, PDI = 4.20.

PTF50: BDT2F-Sn (100 mg, 0.1063 mmol), BDD-Br (40.7 mg, 0.0531 mmol), TFB-Br (19.7 mg, 0.0531 mmol). (80 mg). M_n = 126 kDa, PDI = 4.15.

2. Measurements

The ^{1}H NMR (400 MHz) and ^{13}C NMR (100 MHz) spectra were recorded on Bruker DPX-400 MHz NMR spectrometer in $CDCl_3$. The thermogravimetric analysis (TGA) was performed using a Dupont 9900 analyzer; the samples were measured under N_2 condition and heated from room temperature to 600 °C at a rate of 10 °C min^{-1}. The UV-vis absorption measurements were performed with a Lambda20 (Perkin Elmer) diode array spectrophotometer over 300 ~ 1000 nm. All solutions used in the UV-vis experiments were diluted in *o*-xylene and films were prepared by spin-coating the *o*-xylene solution onto quartz substrates. The Electrochemical cyclic voltammetry (CV) was measured by using an IviumStat instrument and conducted at a scan rate of 50 mV s^{-1} at 25 °C under argon with 0.1 M tetrabutylammonium hexafluorophosphate (Bu_4NPF_6) in acetonitrile as the electrolyte. Donor polymers were deposited onto the working electrode from *o*-xylene solution. All CV measurements were conducted at room temperature with a conventional three-electrode configuration employing a glassy carbon electrode as the working electrode, a saturated calomel electrode (SCE) as the reference electrode, and a Pt wire as the counter electrode. Atomic force microscopy (AFM) images of films were obtained on a Nanoscope IIIa Dimension 3100 operating in ScanAsyst mode. Transmission electron microscopy (TEM) images were obtained on a FEI Tecnai TF20 (Philip) instrument. 2D GIWAXS measurements were conducted at 3C & 9A beamline of the Pohang Accelerator Laboratory (PAL) in Republic of Korea.

3. Device fabrication

Unit cells: All the devices were manufactured with the structure of glass ITO/ZnO NPs/PEIE/photoactive layer/MoO_X/Ag. The pre-patterned ITO substrates were sequentially cleaned with detergent, deionized water, IPA and acetone by ultra-sonication for 15 min each. After drying the ITO substrates and treating the surface with UV ozone for 20 min, the ZnO NPs precursor was spin-coated at 4000 rpm for 45 s onto the ITO surface. After thermal annealing at 100 °C for 10 min in air, the PEIE solution was spun onto the substrates at 5000

rpm for 30 s, which was followed by annealing at 100 °C for 10 min. The thickness of the PEIE layer was approximately 5 nm. And then the substrates were transferred into a nitrogen-filled glovebox for the deposition of photoactive films. The optimal conditions for preparation of the polymer:Y6-BO (weight ratio 1.0:1.2) blend films were found to be spin-coating a total blend concentration of 24 mg mL^{-1} in *o*-xylene at 2500 rpm for 30 s. After thermal annealing at 130 °C for 10 min in a nitrogen-filled glovebox, subsequently, the structure of MoO_X/Ag (10/100 nm) was deposited over the photoactive layer by thermal evaporation in a vacuum chamber with a mask to accomplish the device fabrication. The effective area of one cell was 0.12 cm^2.

Large-area sub-module devices: The pre-patterned large-area ITO substrates were cleaned as per the unit cell above. The pre-cleaned ITO substrates were further treated with UV ozone for 20 min. Then the ZnO NPs solution was bar-coated onto the UV/ozone-treated ITO glass using automatic bar-coating system (PEMS Inc.) with the bar-coating speed of 7 mm s^{-1} and a final thickness of ZnO NPs film was approximately 30 nm. The 15 μL of polymer:Y6-BO solution dissolved in *o*-xylene was then delivered onto the top edge of the ZnO NPs layer, followed by the bar-coating procedure with a speed of 15 mm s^{-1}, under ambient conditions. After drying the photoactive layer, the large-area substrates were transferred to an N_2-filled glove box and heated on a hot plate at a moderate temperature of 80 °C for 30 min. Next, 10 nm of MoO_X and 100 nm of Ag were thermally evaporated onto the photoactive layer under high vacuum using the shadow mask as a hole transport layer and an anode, respectively. The large-area OSC consists 11 stripe pixels of photoactive area and the size of each stripe pixel is 4.95 cm^2 defined by the aperture.

4. Photovoltaic characterization

The OSCs were characterized from *J–V* measurement using Keithley 2400 source meter and a solar simulator (K201 LAB55, McScience), under simulated 100 mW cm^{-2} irradiation from Xe arc lamp with an AM 1.5G filter. Simulator irradiance was characterized using a calibrated spectrometer and the illumination intensity was set using an NREL-certified silicon diode with an integrated KGI optical filter. The EQEs were measured using a spectral measurement system (K3100 IQX, McScience Inc.) which applied monochromatic light from a 100 W Xe arc lamp filtered by an optical chopper. The light intensity dependence of V_{OC} and J_{SC} were performed by setting a series of light intensity and then measured the *J–V* characteristics.

5. Hole and electron-only devices and charge carrier mobility measurements

Hole and electron mobilities were measured using the space charge limited current (SCLC) method, with hole-only device of ITO/poly(3,4-ethylenedioxythiophene):poly(styrenesulfonate) (PEDOT:PSS)/photoactive layer/Au for hole mobility measurement and the electron-only devices used configuration of ITO/ZnO NPs/PEIE/photoactive layer/Ca/Al by taking $J^{0.5}$-V curves in the range of 0 V ~ 5 V. The SCLC mobilities were calculated by MOTT-Gurney equation, which is described by: $J = 9\varepsilon_0\varepsilon_r\mu V^2/8L^3$, where J is the current density, L is the film thickness of photoactive layer, ε_0 is the permittivity of free space (8.85×10^{-12} F m^{-1}), ε_r is the relative dielectric constant of the transport medium, μ is the hole or electron mobility, V is the internal voltage in the device and $V = V_{appl} - V_r - V_{bi}$, where V_{appl} is the applied voltage to the device, V_r is the voltage drop due to contact resistance and series resistance across the electrodes, and V_{bi} is the built-in voltage due to the relative work function difference of the two electrodes.

6. Grazing-incidence wide-angle X-ray scattering (GIWAXS) analysis

The GIWAXS measurements were performed at 3C & 9A beamline of the Pohang Accelerator Laboratory (PAL) in Republic of Korea. The X-ray radiation beam energy was 11.09 keV (wavelength: 1.11794 Å) with a sample-to-detector distance of approximately 221 mm. The incidence angle for the X-ray beam was set to 0.11° ~ 0.12° to characterize the optimized neat and blend films. The GIWAXS patterns were collected from a 2D CCD detector (Rayonix SX165). The measured data was analyzed using the Igor-Pro software package. The GIWAXS samples were prepared by employing Si-wafer/ZnO NPs/PEIE/thin films as fabricated in the optimized device conditions.

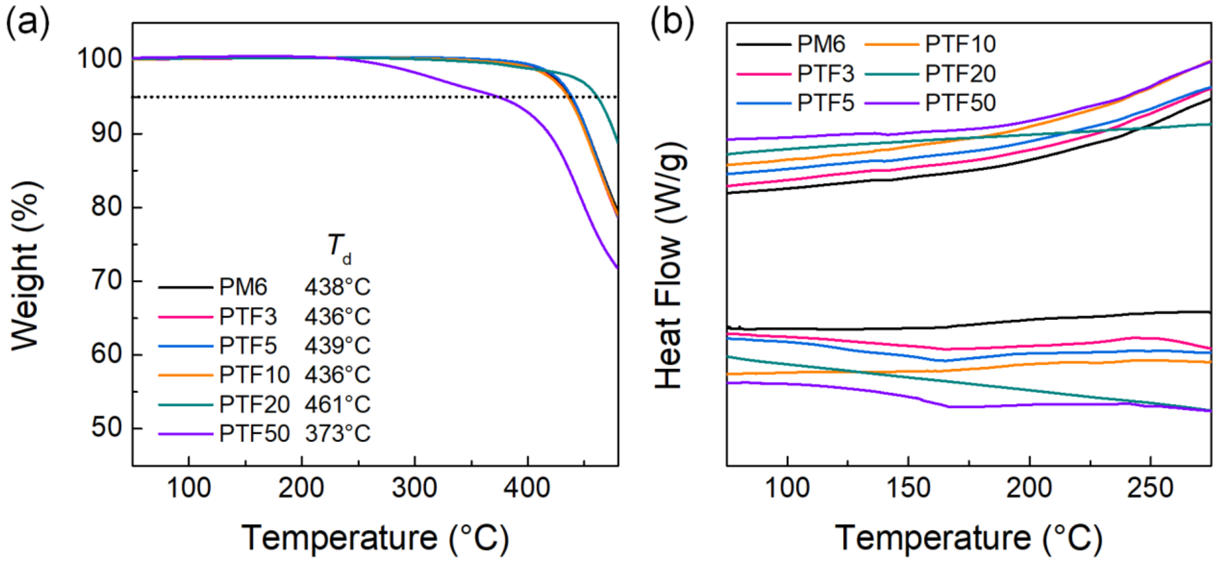

Figure S1. (a) Thermogravimetric analysis (TGA) traces and (b) Differential scanning calorimetry (DSC) thermograms of PM6 and terpolymers.

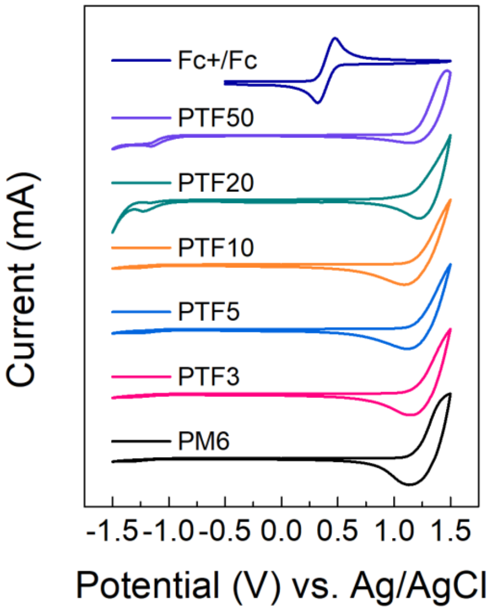

Figure S2. Cyclic voltammogram (CV) characteristics of PM6 and terpolymers.

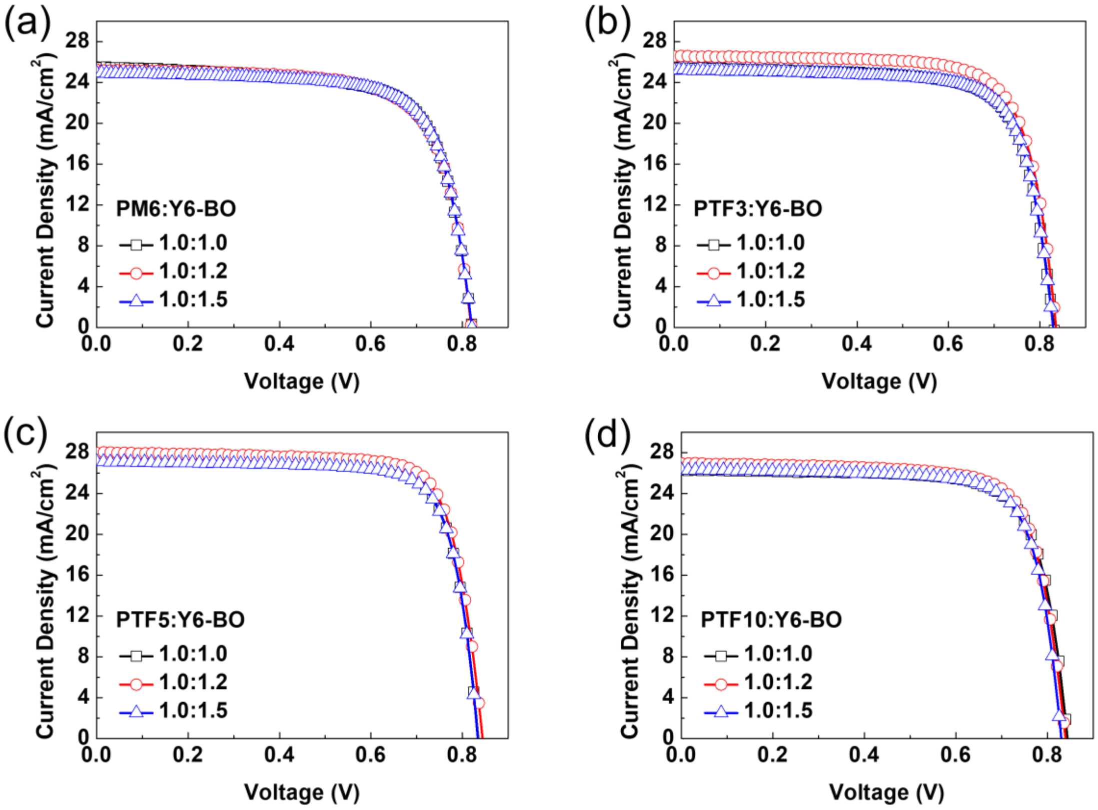

Figure S3. *J*-*V* characteristics of polymer donor:Y6-BO OSCs with different weight ratios of (a) PM6, (b) PTF3, (b) PTF5, and (b) PTF10 under AM 1.5G illumination.

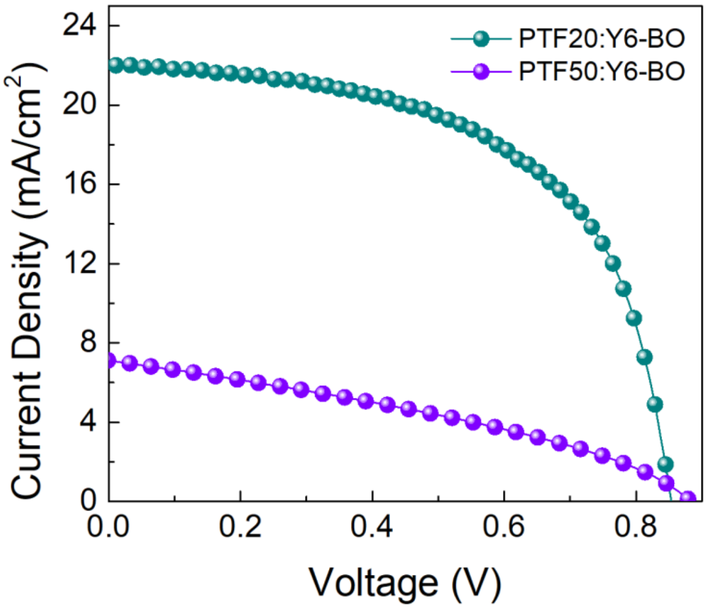

Figure S4. Best *J*–*V* characteristic curves of PTF20:Y6-BO and PTF50:Y6-BO-based OSCs.

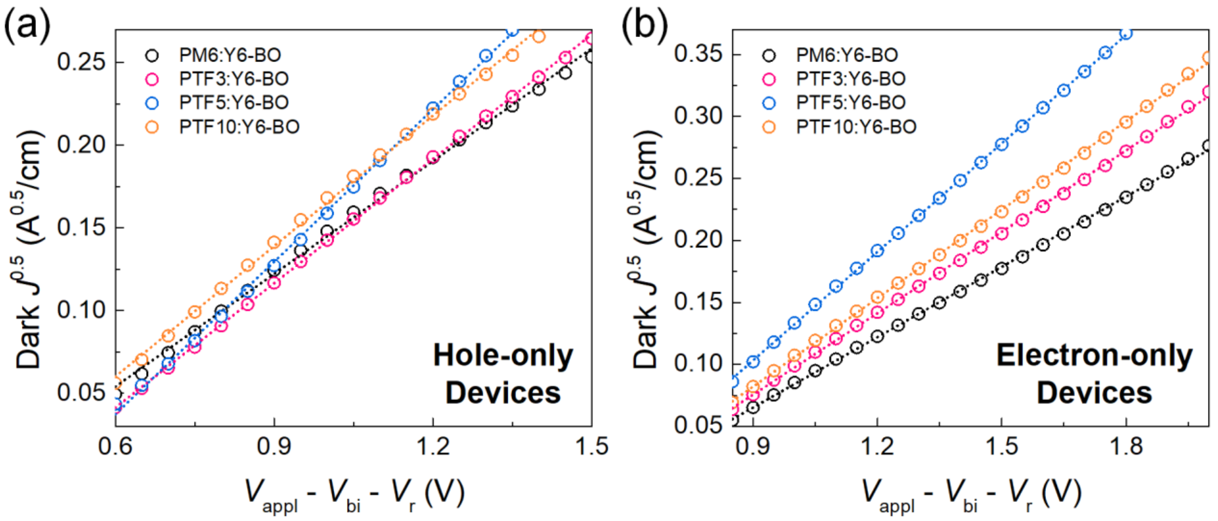

Figure S5. SCLC characteristic curves of (a) hole-only and (b) electron-only devices based on donor polymer:Y6-BO photoactive layers.

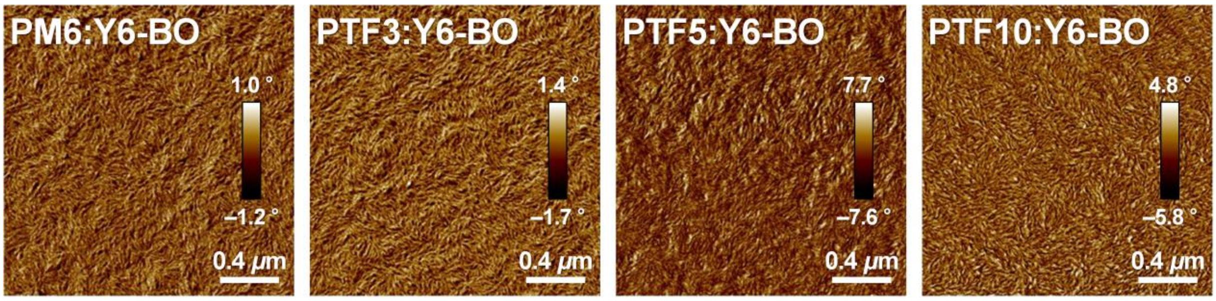

Figure S6. AFM phase images of donor polymer:Y6-BO blend films.

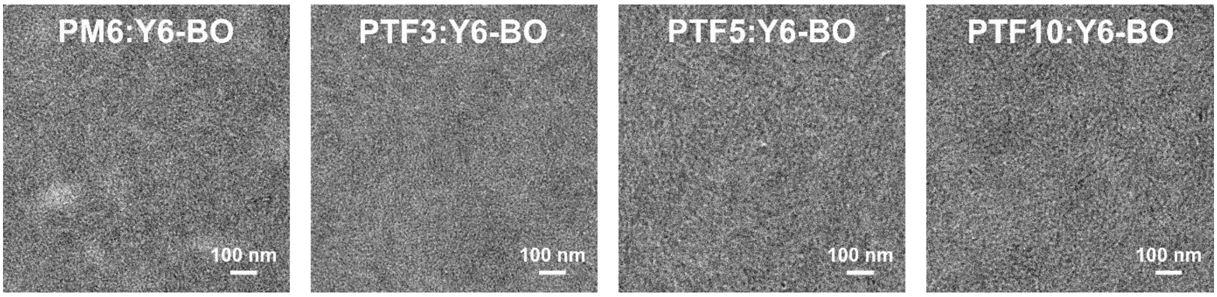

Figure S7. High-magnification TFM images of donor polymer:Y6-BO blend films.

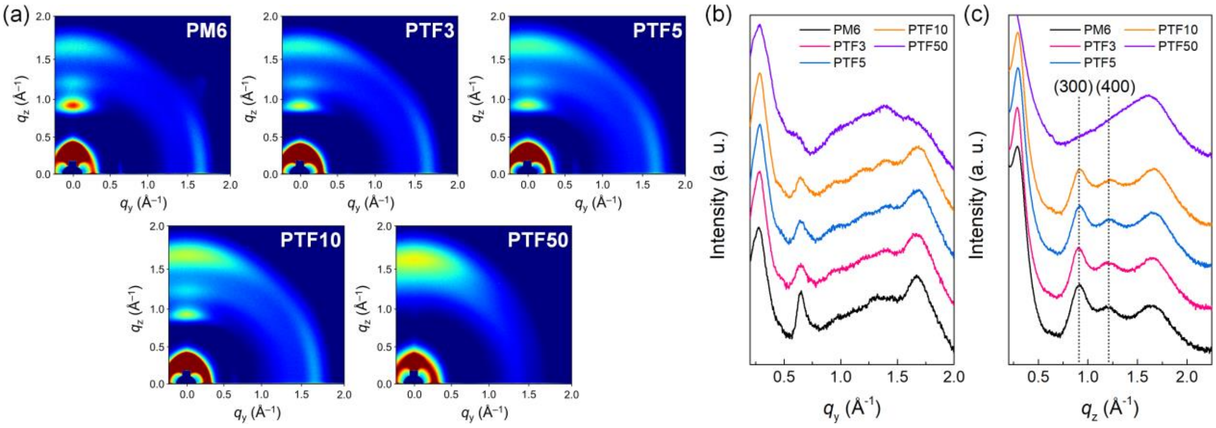

Figure S8. (a) 2D GIWAXS scattering pattern images of PM6 and terpolymers. Line-cut profiles for (b) in-plane (IP) direction and (c) out-of-plane (OOP) directions, respectively.

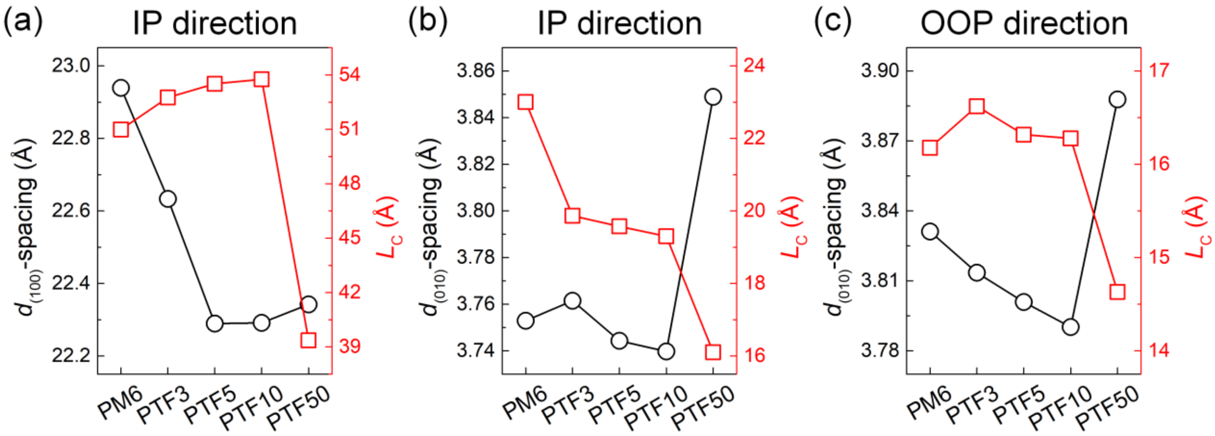

Figure S9. *d*-spacing and L_C values of neat films for (a) lamellar stacking and (b) *π*–*π* stacking along the IP direction. (c) *d*-spacing and L_C values of neat films for *π*–*π* stacking along the OOP direction.

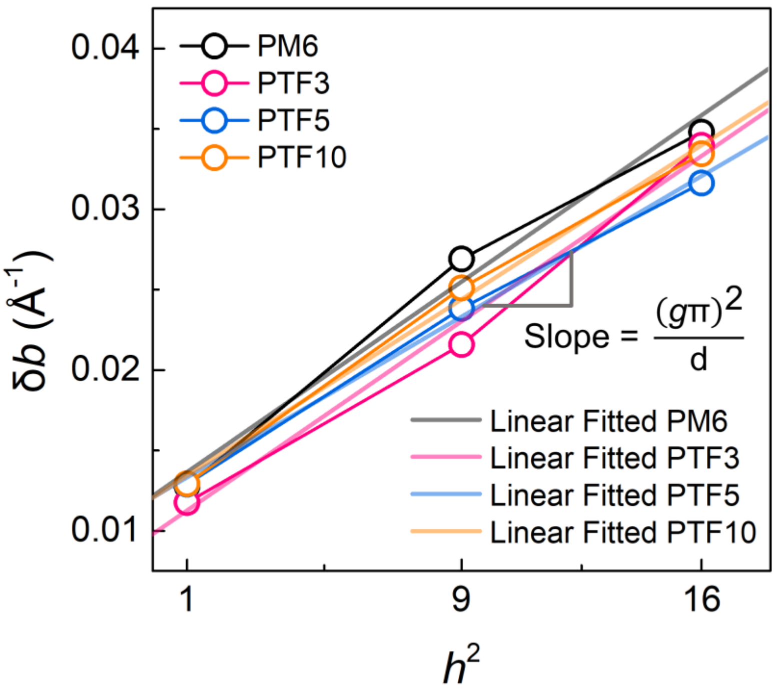

Figure S10. Hosemann plots (δb–h^2) of PM6 and terpolymer neat films extracted from line-cut profiles for (h00) of GIWAXS in the OOP direction.

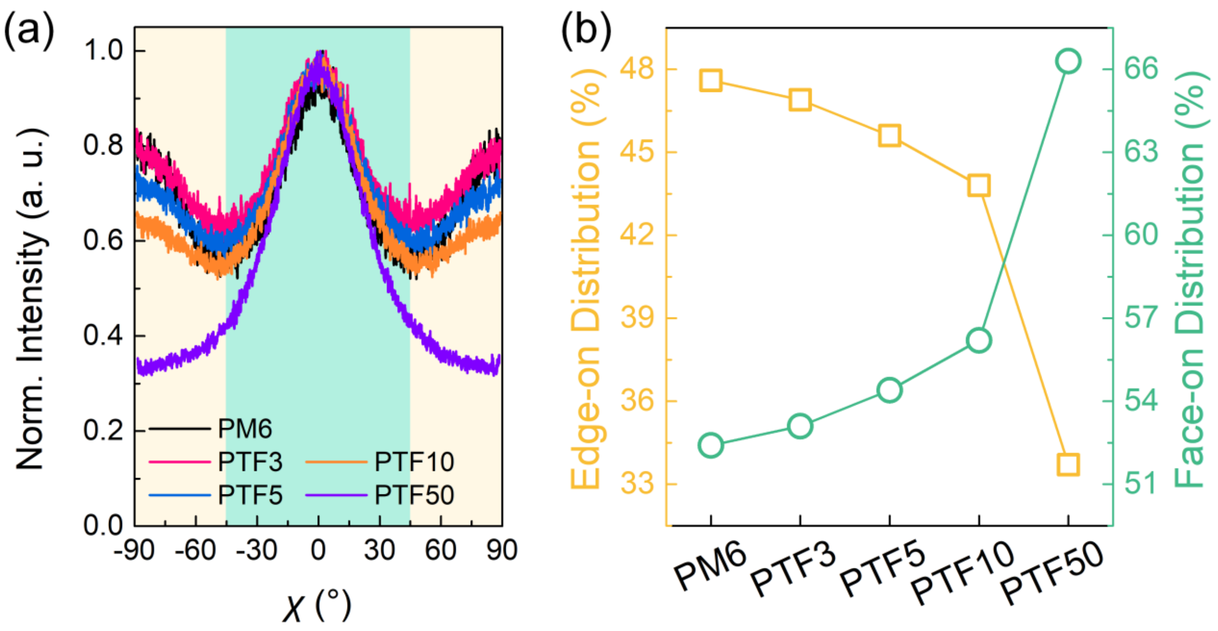

Figure S11. (a) Azimuthal cut plots of (010) scattering peaks for PM6 and terpolymer neat films. (b) Face-on and edge-on orientation distribution of each neat film.

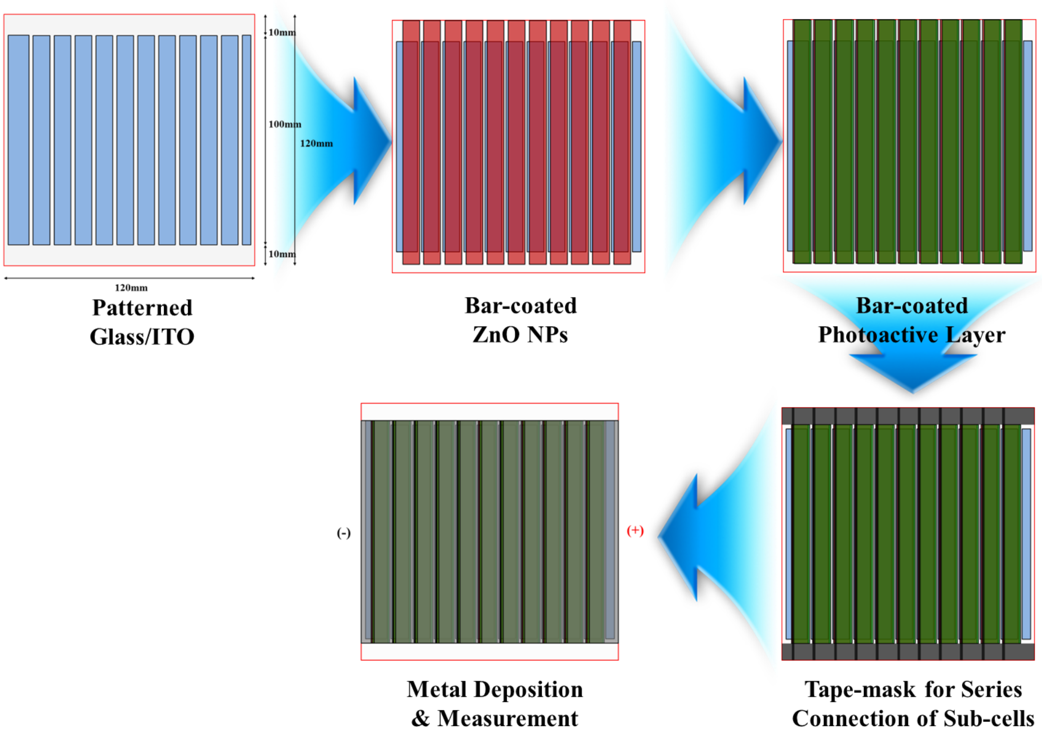

Figure S12. Schematic illustration of the large-area OSC fabrication procedure using the D-bar coater.

시 험 결 과

성적서 번호 : KIER-210126007호
페이지(5) / (총6)

[첨부2]

한국화학연구원
유기 태양전지 미니모듈

모델명/식별번호 : OPV mini-module #1
시험 일자 : 2021.02.02.
솔라시뮬레이터 : WACOM, WXS-155S-L2 (Class-AAA)
기준 태양전지 : KIER-PS-FD #2
시험 조건 : STC [AM1.5G, 100 mW/cm², (25.0 ± 1.0) °C]

소자 면적 : 54.406 cm² (의뢰자 지정 면적)
시료 유형 : 유기 태양전지 미니모듈 (유리판 기반)

한국에너지기술연구원
KOREA INSTITUTE OF ENERGY RESEARCH

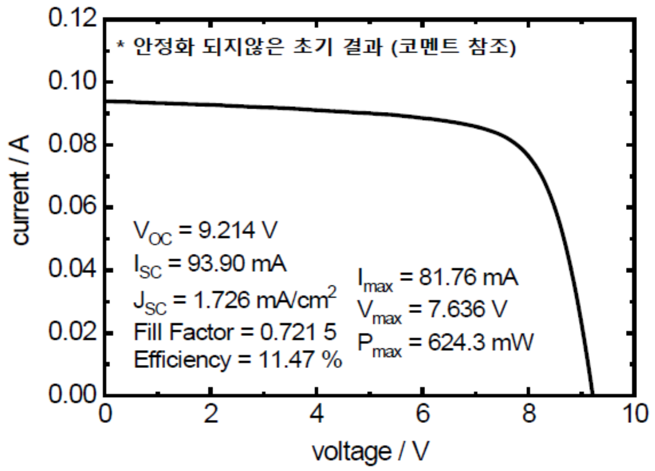

시험자: 이상민

(34129) 대전광역시 유성구 가정로 152 한국에너지기술연구원 태양광연구단
Tel : +82-42-860-3182, e-mail : notask@kier.re.kr

Figure S13. *J–V* curve of the large-area OSC based on PTF5:Y6-BO photoactive layer certified by Korea Institute of Energy Research (KIER).

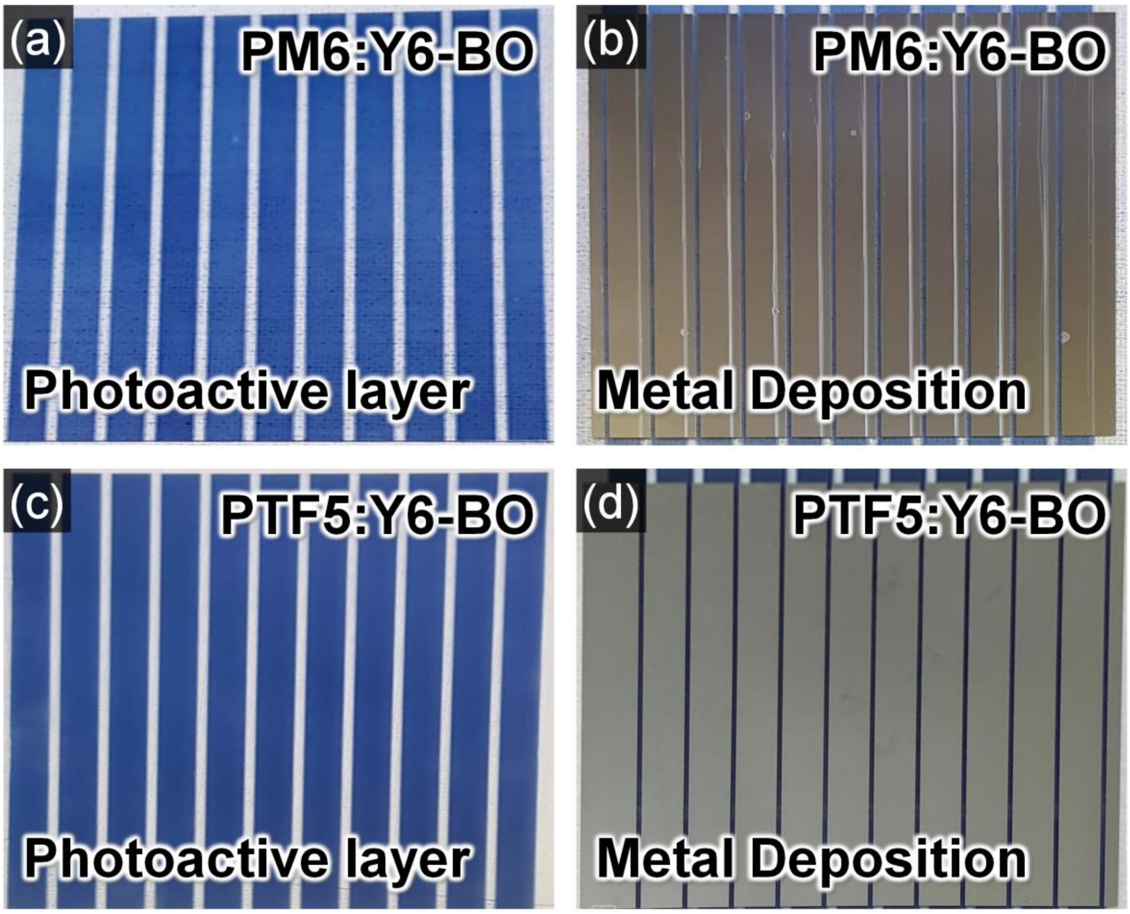

Figure S14. Images (a) after coating the PM6:Y6-BO photoactive material and (b) after metal deposition on the PM6:Y6-BO photoactive layer. Images (a) after coating the PTF5:Y6-BO photoactive material and (b) after metal deposition on the PTF5:Y6-BO photoactive layer.

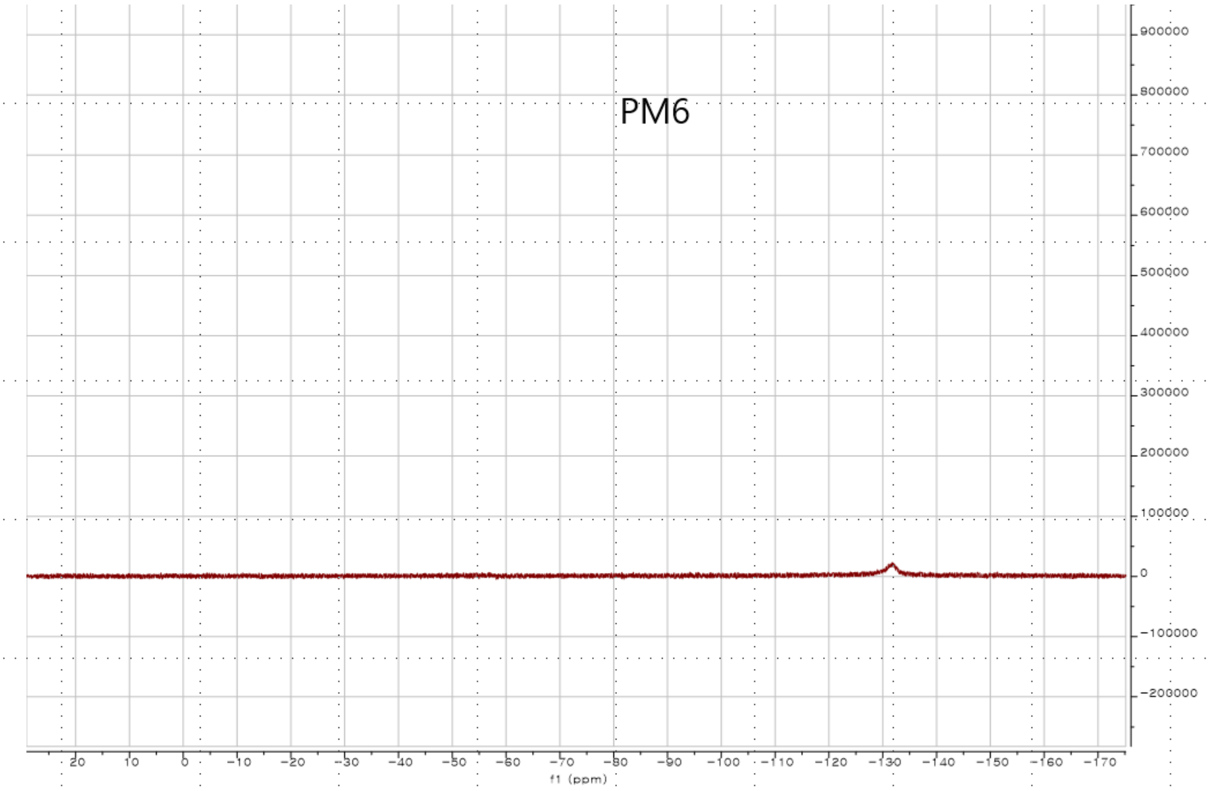

Figure S15. ^{19}F NMR spectrum of PM6 polymer donor.

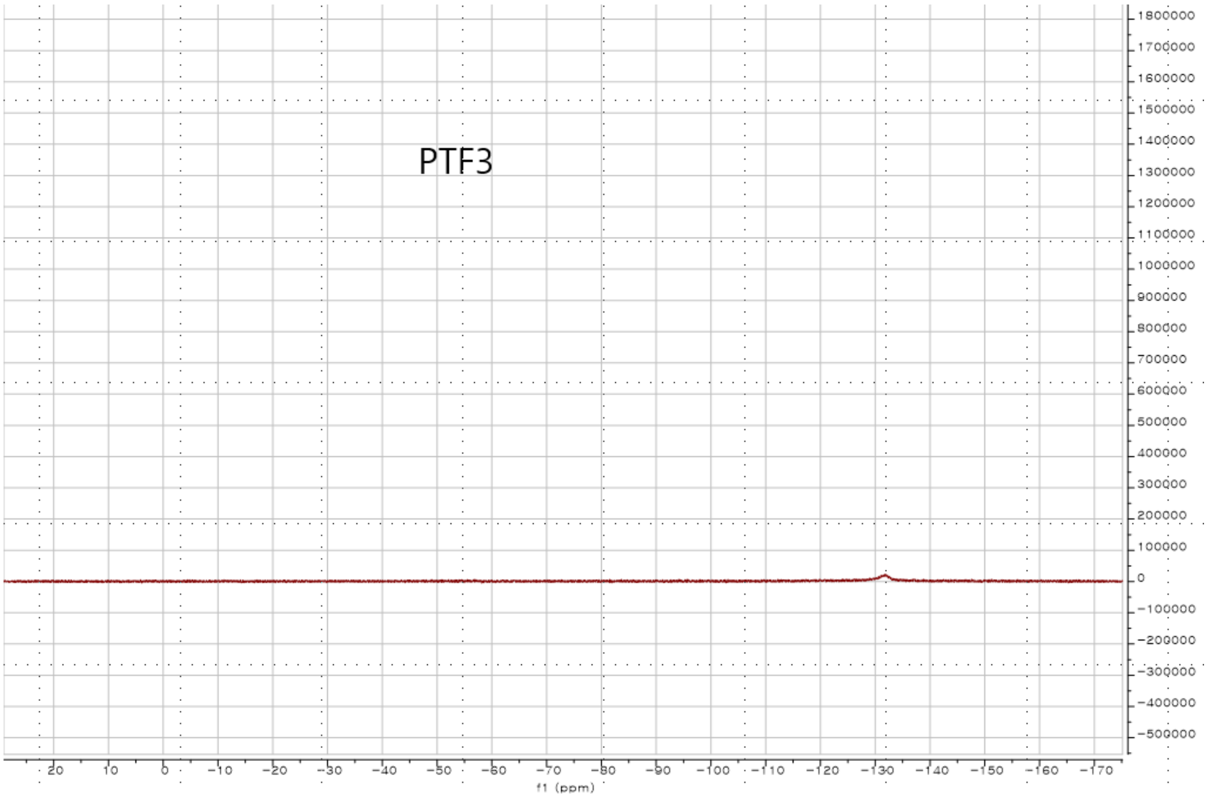

Figure S16. ^{19}F NMR spectrum of PTF3 polymer donor.

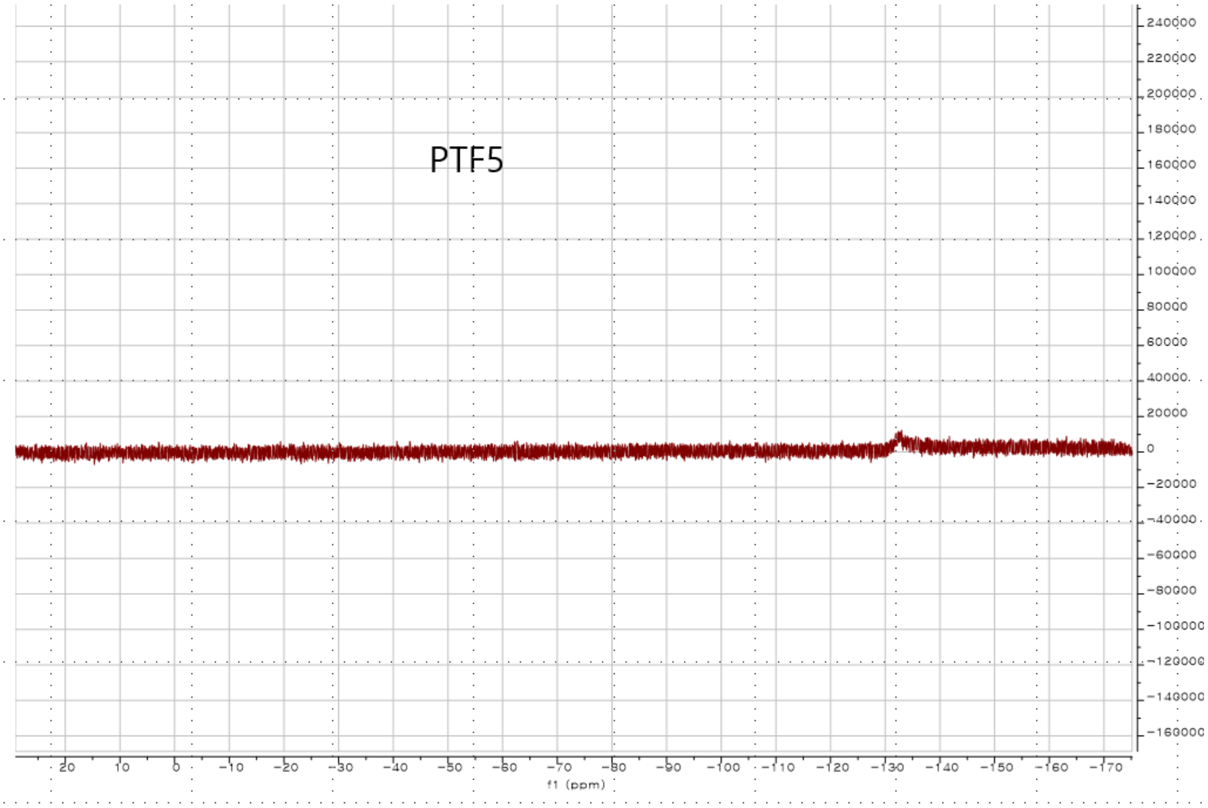

Figure S17. ^{19}F NMR spectrum of PTF5 polymer donor.

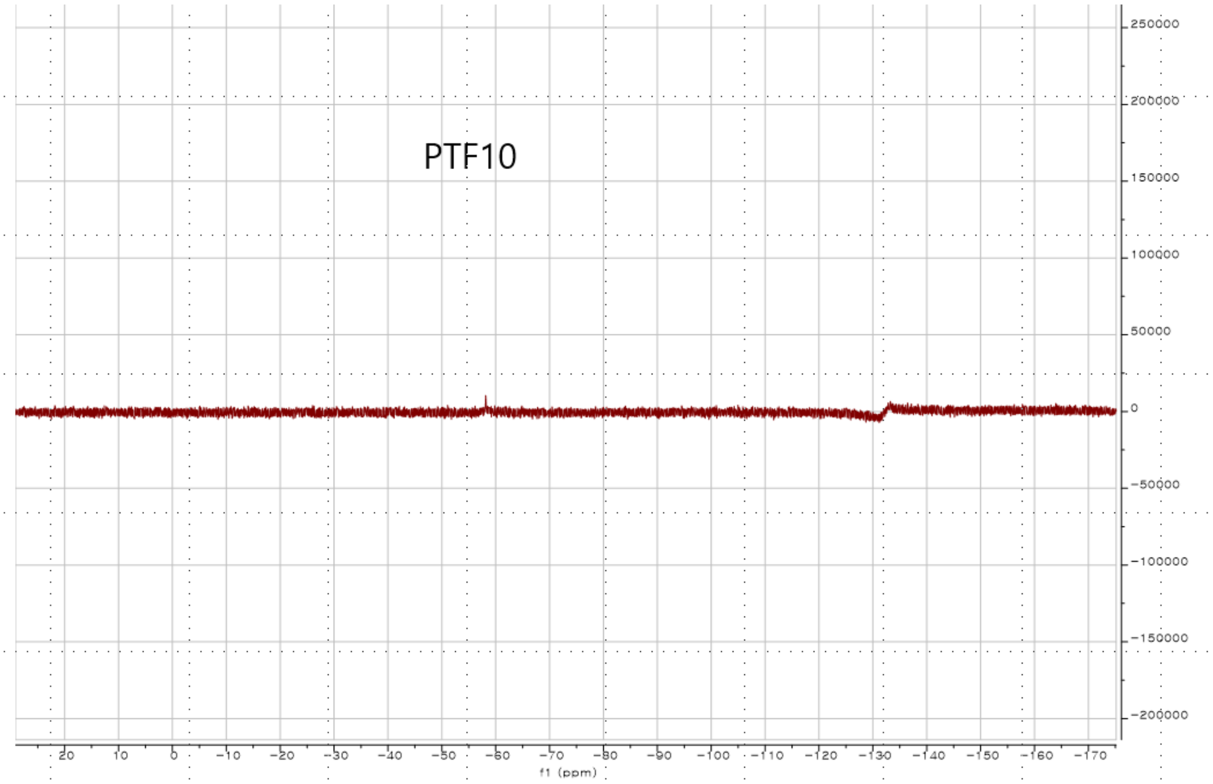

Figure S18. ^{19}F NMR spectrum of PTF10 polymer donor.

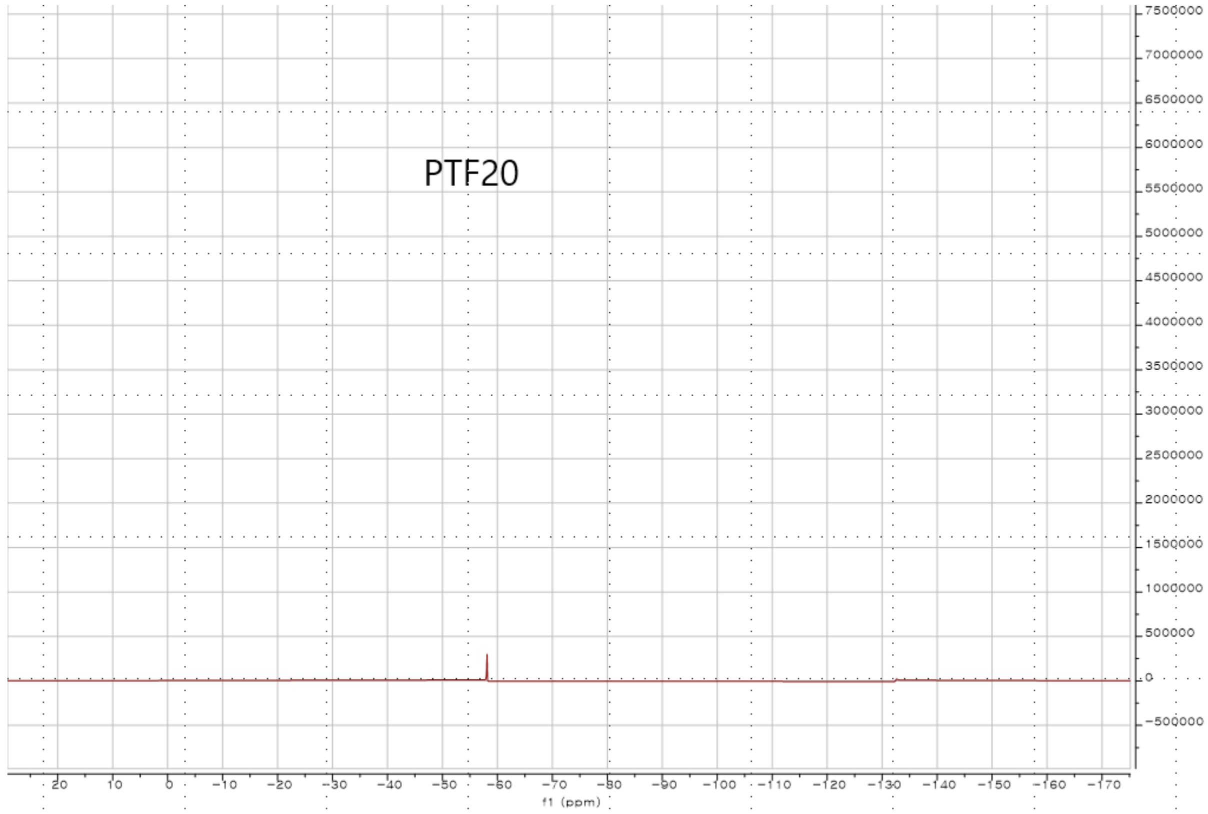

Figure S19. ^{19}F NMR spectrum of PTF20 polymer donor.

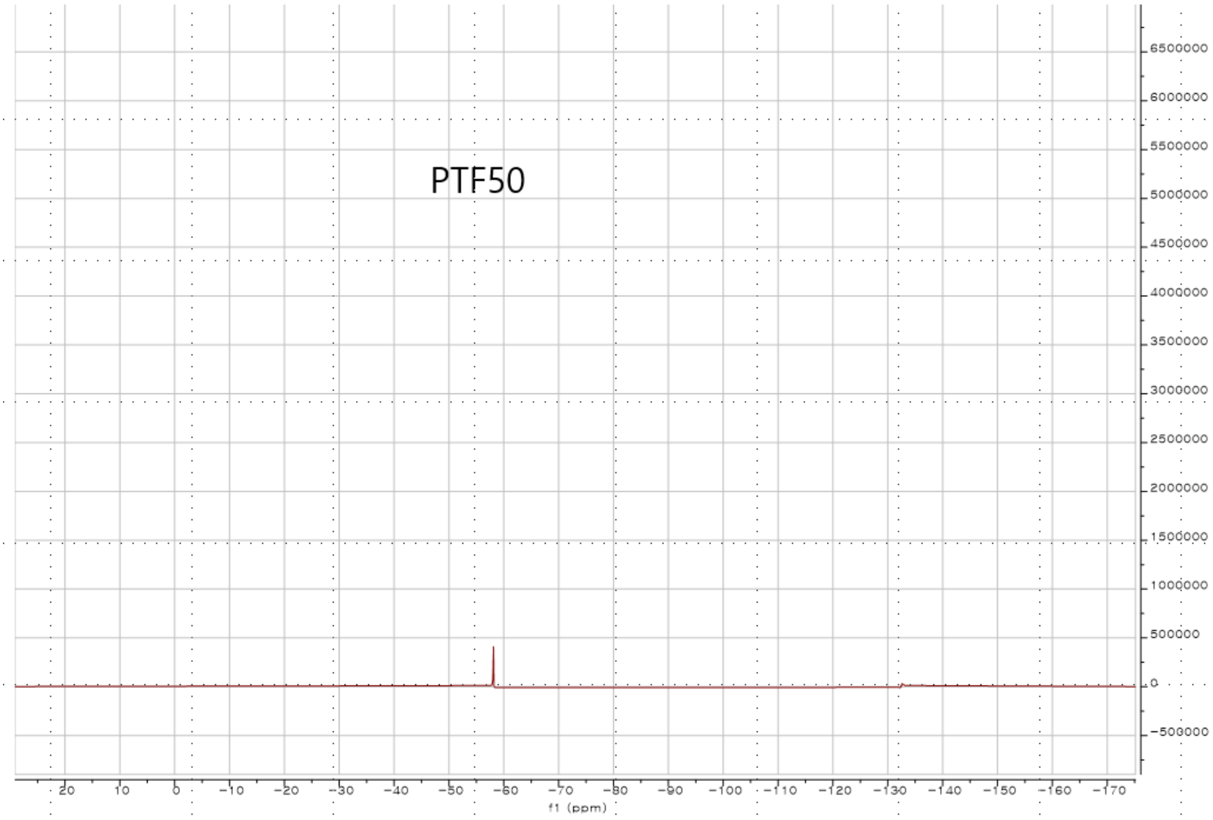

Figure S20. ^{19}F NMR spectrum of PTF50 polymer donor.

Table S1. Photovoltaic parameters of OSCs with different weight ratios of polymer donor:Y6-BO photoactive layer under an illumination of air mass 1.5 global (AM 1.5G), 100 mW/cm^2.

**Polymer donor**[a)]	**Weight ratio**	V_{OC} **[V]**	J_{SC} **[mA/cm^2]**	***FF*** **[%]**	**PCE [%]**
PM6	1.0:1.0	0.82	25.5	70.3	14.6 (14.4±0.21)
	1.0:1.2	0.82	25.8	71.3	15.1 (14.9±0.26)
	1.0:1.5	0.82	24.9	72.2	14.7 (14.5±0.16)
PTF3	1.0:1.0	0.83	25.5	73.4	15.4 (15.3±0.18)
	1.0:1.2	0.83	26.5	74.0	16.3 (16.0±0.36)
	1.0:1.5	0.83	25.2	74.2	15.5 (15.4±0.12)
PTF5	1.0:1.0	0.84	27.5	76.9	17.8 (17.6±0.17)
	1.0:1.2	0.84	28.0	77.2	18.2 (17.8±0.34)
	1.0:1.5	0.83	27.1	77.1	17.4 (17.1±0.22)
PTF10	1.0:1.0	0.84	26.3	74.7	16.5 (16.3±0.19)
	1.0:1.2	0.84	27.0	75.1	17.02 (16.8±0.35)
	1.0:1.5	0.83	26.3	75.8	16.6 (16.5±0.13)

[a)]Inverted device architecture is ITO/ZnO NPs/PEIE/photoactive layer(d = 130nm)/MoO_X/Ag. Photoactive materials are dissolved in *o*-xylene and annealed at 130C °C for 10 min.
The values in parenthesis are average photovoltaic properties obtained from over 5 devices.

Table S2. Detailed photovoltaic parameters of OSCs based on PTF20:Y6-BO and PTF50:Y6-BO photoactive layers processed with *o*-xylene under an illumination of AM 1.5G at 100 mW cm^{-2}.

Photoactive layer[a)]	V_{OC} [V]	J_{SC} [mA cm^{-2}]	FF [%]	PCE [%]
PTF20:Y6-BO	0.86	22.1	57.0	10.8
PTF50:Y6-BO	0.88	7.11	35.0	2.20

[a)]Inverted device architecture is ITO/ZnO NPs/PEIE/Photoactive layer ($d \approx 130$ nm)/MoO_X/Ag. Photoactive materials were dissolved in *o*-xylene and annealed at 120 °C for 10 min.

Table S3. Summary of contact angles for water and diiodomethane, surface energy, and Flory-Huggins interaction parameters of Y6-BO and donor polymers.

Film	Contact angle		γ [mN m^{-1}]	χ
	θ_{water} [°]	θ_{DIM} [°]		
Y6-BO	94.2	38.4	40.4	-
PM6	93.9	46.8	36.1	0.12
PTF3	94.8	47.5	35.5	0.16
PTF5	95.2	48.4	35.2	0.18
PTF10	96.7	50.7	33.9	0.29
PTF50	98.2	54.4	32.4	0.45

Table S4. Summary of crystallographic parameters for donor polymer and Y6-BO neat films.

Film	In-plane (IP)				Out-of-plane (OOP)			
	$d_{(100)}$ [Å]	$L_{C(100)}$ [Å]	$d_{(010)}$ [Å]	$L_{C(010)}$ [Å]	$d_{(100)}$ [Å]	$L_{C(100)}$ [Å]	$d_{(010)}$ [Å]	$L_{C(010)}$ [Å]
PM6	22.9	51.0	3.75	23.0	21.5	78.3	3.83	16.2
PTF3	22.6	52.8	3.76	19.9	21.9	85.0	3.81	16.6
PTF5	22.3	53.5	3.74	19.6	21.3	77.6	3.80	16.3
PTF10	22.4	53.8	3.74	19.3	21.4	77.4	3.79	16.3
PTF50	22.3	39.4	3.85	16.1	-	-	3.89	14.6
Y6-BO	-	-	-	-	-	-	3.62	26.8

Table S5. Summary of (*h*00) scattering peaks and the paracrystalline disorder parameter (g) for PM6 and terpolymers along the OOP direction.

Polymer	OOP direction								g [%]
	$d_{(100)}$ [Å]	$L_{C(100)}$ [Å]	$d_{(300)}$ [Å]	$L_{C(300)}$ [Å]	$d_{(100)}/d_{(300)}$	$d_{(400)}$ [Å]	$L_{C(400)}$ [Å]	$d_{(100)}/d_{(400)}$	
PM6	21.5	78.3	6.88	37.2	3.13	5.28	28.7	4.08	5.68
PTF3	21.9	85.0	6.97	46.3	3.13	5.18	29.4	4.22	5.71
PTF5	21.3	77.6	6.89	41.9	3.10	5.09	31.6	4.19	5.20
PTF10	21.4	77.4	6.89	39.8	3.11	5.09	29.9	4.21	5.53

Table S6. Orientation distribution of PM6 and terpolymer neat films extracted from (010) scattering peaks.

Polymer	Edge-on [%]	Face-on [%]
PM6	47.6	52.4
PTF3	46.9	53.1
PTF5	45.6	54.4
PTF10	43.8	56.2
PTF50	33.7	66.3

Table S7. Summary of crystallographic parameters for donor polymer:Y6-BO blend films.

Blend film	IP		OOP	
	$d_{(100)}$ [Å]	$L_{C(100)}$ [Å]	$d_{(010)}$ [Å]	$L_{C(010)}$ [Å]
PM6:Y6-BO	21.8	96.2	3.68	24.1
PTF3:Y6-BO	21.8	126	3.66	22.6
PTF5:Y6-BO	21.7	128	3.65	22.8
PTF10:Y6-BO	21.8	123	3.66	21.9
PTF50:Y6-BO	21.5	84.2	3.63	26.7

Table S8. Summary of photoactive area and PCE of representative binary OSCs based on unit and mini/sub-module devices.

Device	Photoactive area [cm^2]	PCE [%]	Ref.
Unit	0.04	17.48	[3]
	0.08	17.98	[4]
	0.04	17.50	[5]
	0.03152	18.32	[6]
	0.04	18.74	[7]
	0.05	17.98	[8]
	0.056	17.12	[9]
	0.04	18.20	[10]
	0.09	17.40	[11]
	0.1350	17.38	[12]
	0.04	18.00	[13]
	0.0478	18.86	[14]
	0.059	17.42	[15]
	0.050	16.88	[16]
	0.045	17.20	[17]
	0.09	17.92	[18]
	1.0	16.10	[19]
	0.04572	18.67	[20]
	1.0	16.86	[8]
	1.0	15.20	[21]
Mini/Sub-module	12.6	16.86	[22]
	80	15.20	[23]
	20.4	10.13	[24]
	10.8	9.80	[25]
	11.52	11.86	[26]
	58.5	9.03	[27]
	21	5.1	[28]
	30	8.8	[29]
	59.52	4.40	[30]
	58.5	7.74	[31]
	16.6	7.40	[32]
	10.8	2.70	[33]

References

[1] M. Zhang, X. Guo, W. Ma, H. Ade, J. Hou, *Adv. Mater.* **2015**, *27*, 4655.

[2] D. Qian, L. Ye, M. Zhang, Y. Liang, L. Li, Y. Huang, X. Guo, S. Zhang, Z. a. Tan, J. Hou, *Macromolecules* **2012**, *45*, 9611.

[3] Y. Zhang, K. Liu, J. Huang, X. Xia, J. Cao, G. Zhao, P. W. K. Fong, Y. Zhu, F. Yan, Y. Yang, X. Lu, G. Li, *Nat. Commun.* **2021**, *12*, 4815.

[4] Y. Su, L. Zhang, Z. Ding, Y. Zhang, Y. Wu, Y. Duan, Q. Zhang, J. Zhang, Y. Han, Z. Xu, R. Zhang, K. Zhao, S. Liu, *Adv.Energy Mater.* **2022**, *12*, 2103940.

[5] L. Wang, Q. An, L. Yan, H.-R. Bai, M. Jiang, A. Mahmood, C. Yang, H. Zhi, J.-L. Wang, *Energy Environ. Sci.* **2022**, *15*, 320.

[6] P. Bi, S. Zhang, Z. Chen, Y. Xu, Y. Cui, T. Zhang, J. Ren, J. Qin, L. Hong, X. Hao, J. Hou, *Joule* **2021**, *5*, 2408.

[7] X. Xu, L. Yu, H. Meng, L. Dai, H. Yan, R. Li, Q. Peng, *Adv. Funct. Mater.* **2022**, *32*, 2108797.

[8] R. Sun, T. Wang, X. Yang, Y. Wu, Y. Wang, Q. Wu, M. Zhang, C. J. Brabec, Y. Li, J. Min, *Nat. Energy* **2022**, *7*, 1087.

[9] J. Qin, Q. Yang, J. Oh, S. Chen, G. O. Odunmbaku, N. A. N. Ouedraogo, C. Yang, K. Sun, S. Lu, *Adv. Sci.* **2022**, *9*, 2105347.

[10] S. Li, Q. Fu, L. Meng, X. Wan, L. Ding, G. Lu, G. Lu, Z. Yao, C. Li, Y. Chen, *Angew. Chem. Int. Ed.* **2022**, *61*, e202207397.

[11] Z. Abbas, S. U. Ryu, M. Haris, C. E. Song, H. K. Lee, S. K. Lee, W. S. Shin, T. Park, J.-C. Lee, *Nano Energy* **2022**, *101*, 107574.

[12] S. Rasool, J. W. Kim, H. W. Cho, Y. J. Kim, D. C. Lee, C. B. Park, W. Lee, O. H. Kwon, S. Cho, J. Y. Kim, *Adv. Energy Mater.* **2023**, *13*, 2203452.

[13] J. Wang, Y. Cui, Y. Xu, K. Xian, P. Bi, Z. Chen, K. Zhou, L. Ma, T. Zhang, Y. Yang, Y. Zu, H. Yao, X. Hao, L. Ye, J. Hou, *Adv. Mater.* **2022**, *34*, 2205009.

[14] C. He, Y. Pan, G. Lu, B. Wu, X. Xia, C. Q. Ma, Z. Chen, H. Zhu, X. Lu, W. Ma, L. Zuo, H. Chen, *Adv. Mater.* **2022**, *34*, 2203379.

[15] K. Jiang, J. Zhang, Z. Peng, F. Lin, S. Wu, Z. Li, Y. Chen, H. Yan, H. Ade, Z. Zhu, A. K. Jen, *Nat. Commun.* **2021**, *12*, 468.

[16] L. Zhu, M. Zhang, G. Zhou, T. Hao, J. Xu, J. Wang, C. Qiu, N. Prine, J. Ali, W. Feng, X. Gu, Z. Ma, Z. Tang, H. Zhu, L. Ying, Y. Zhang, F. Liu, *Adv. Energy Mater.* **2020**, *10*, 1904234.

[17] H. Fu, W. Gao, Y. Li, F. Lin, X. Wu, J. H. Son, J. Luo, H. Y. Woo, Z. Zhu, A. K.-Y. Jen, *Small Methods* **2020**, *4*, 2000687.

[18] E. Moustafa, M. Méndez, J. G. Sánchez, J. Pallarès, E. Palomares, L. F. Marsal, *Adv. Energy Mater.* **2023**, *13*, 2203241.

[19] Y. Cho, Z. Sun, K. M. Lee, G. Zeng, S. Jeong, S. Yang, J. E. Lee, B. Lee, S.-H. Kang, Y. Li, Y. Li, S. K. Kwak, C. Yang, *ACS Energy Lett.* **2022**, *8*, 96.

[20] T. Chen, S. Li, Y. Li, Z. Chen, H. Wu, Y. Lin, Y. Gao, M. Wang, G. Ding, J. Min, Z. Ma, H. Zhu, L. Zuo, H. Chen, *Adv. Mater.* **2023**, 2300400. DOI: 10.1002/adma.202300400.

[21] U. Würfel, J. Herterich, M. List, J. Faisst, M. F. M. Bhuyian, H.-F. Schleiermacher, K. T. Knupfer, B. Zimmermann, *Sol. RRL* **2021**, *5*, 2000802.

[22] W. Zhao, Y. Zhang, S. Zhang, S. Li, C. He, J. Hou, *J. Mater. Chem. C* **2019**, *7*, 3206.

[23] Y. W. Han, S. J. Jeon, H. S. Lee, H. Park, K. S. Kim, H. W. Lee, D. K. Moon, *Adv. Energy Mater.* **2019**, *9*, 1902065.

[24] C.-Y. Liao, Y. Chen, C.-C. Lee, G. Wang, N.-W. Teng, C.-H. Lee, W.-L. Li, Y.-K. Chen, C.-H. Li, H.-L. Ho, P. H.-S. Tan, B. Wang, Y.-C. Huang, R. M. Young, M. R. Wasielewski, T. J. Marks, Y.-M. Chang, A. Facchetti, *Joule* **2020**, *4*, 189.

[25] C.-Y. Tsai, Y.-H. Lin, Y.-M. Chang, J.-C. Kao, Y.-C. Liang, C.-C. Liu, J. Qiu, L. Wu, C.-Y. Liao, H.-S. Tan, Y.-C. Chao, S.-F. Horng, H.-W. Zan, H.-F. Meng, F. Li, *Sol. Energy Mater. Sol. Cells* **2020**, *218*, 110762.

[26] R. Sun, Q. Wu, J. Guo, T. Wang, Y. Wu, B. Qiu, Z. Luo, W. Yang, Z. Hu, J. Guo, M. Shi, C. Yang, F. Huang, Y. Li, J. Min, *Joule* **2020**, *4*, 407.

[27] S. H. Park, S. Park, S. Lee, J. Kim, H. Ahn, B. J. Kim, B. Chae, H. J. Son, *Nano Energy* **2020**, *77*, 105147.

[28] L. Mao, L. Sun, B. Luo, Y. Jiang, Y. Zhou, *J. Mater. Chem. A* **2018**, *6*, 5817.

[29] Y. W. Han, H. S. Lee, D. K. Moon, *ACS Appl. Mater. Interfaces* **2021**, *13*, 19085.

[30] S. Strohm, F. Machui, S. Langner, P. Kubis, N. Gasparini, M. Salvador, I. McCulloch, H. J. Egelhaaf, C. J. Brabec, *Energy Environ. Sci.* **2018**, *11*, 2225.

[31] S. H. Park, S. Park, D. Kurniawan, J. G. Son, J. H. Noh, H. Ahn, H. J. Son, *Chem. Mater.* **2020**, *32*, 3469.

[32] S. Hong, H. Kang, G. Kim, S. Lee, S. Kim, J. H. Lee, J. Lee, M. Yi, J. Kim, H. Back, J. R. Kim, K. Lee, *Nat. Commun.* **2016**, *7*, 10279.

[33] Y. Q. Wong, H.-F. Meng, H. Y. Wong, C. S. Tan, C.-Y. Wu, P.-T. Tsai, C.-Y. Chang, S.-F. Horng, H.-W. Zan, *Org. Electron.* **2017**, *43*, 196.
